# Schizotypy-Related Magnetization of Cortex in Healthy Adolescence Is Colocated With Expression of Schizophrenia-Related Genes

**DOI:** 10.1016/j.biopsych.2019.12.005

**Published:** 2020-08-01

**Authors:** Rafael Romero-Garcia, Jakob Seidlitz, Kirstie J. Whitaker, Sarah E. Morgan, Peter Fonagy, Raymond J. Dolan, Peter B. Jones, Ian M. Goodyer, John Suckling, NSPN Consortium, Edward Bullmore, Raymond Dolan, Ian Goodyer, Peter Fonagy, Peter Jones, Matilde Vaghi, Michael Moutoussis, Tobias Hauser, Sharon Neufeld, Rafael Romero-Garcia, Michelle St Clair, Kirstie Whitaker, Becky Inkster, Gita Prabhu, Cinly Ooi, Umar Toseeb, Barry Widmer, Junaid Bhatti, Laura Villis, Ayesha Alrumaithi, Sarah Birt, Aislinn Bowler, Kalia Cleridou, Hina Dadabhoy, Emma Davies, Ashlyn Firkins, Sian Granville, Elizabeth Harding, Alexandra Hopkins, Daniel Isaacs, Janchai King, Danae Kokorikou, Christina Maurice, Cleo McIntosh, Jessica Memarzia, Harriet Mills, Ciara O’Donnell, Sara Pantaleone, Jenny Scott, Pasco Fearon, John Suckling, Anne-Laura van Harmelen, Rogier Kievit, Petra Vértes, Petra E. Vértes, Edward T. Bullmore

**Affiliations:** a Department of Psychiatry, University of Cambridge, Cambridge, United Kingdom; b Alan Turing Institute, London, United Kingdom; c Research Department of Clinical, Educational and Health Psychology, London, United Kingdom; d Max Planck UCL Centre for Computational Psychiatry and Ageing Research, University College London, London, United Kingdom; e Wellcome Trust Centre for Neuroimaging, UCL Queen Square Institute of Neurology, University College London, London, United Kingdom; f School of Mathematical Sciences, Queen Mary University of London, London, United Kingdom; g Cambridgeshire and Peterborough NHS Foundation Trust, Huntingdon, United Kingdom

**Keywords:** Adolescence, Allen Human Brain Atlas, Development, Fast-spiking GABAergic interneurons, Multiparameter MRI mapping, Myelination, Schizophrenia

## Abstract

**Background:**

Genetic risk is thought to drive clinical variation on a spectrum of schizophrenia-like traits, but the underlying changes in brain structure that mechanistically link genomic variation to schizotypal experience and behavior are unclear.

**Methods:**

We assessed schizotypy using a self-reported questionnaire and measured magnetization transfer as a putative microstructural magnetic resonance imaging marker of intracortical myelination in 68 brain regions in 248 healthy young people (14–25 years of age). We used normative adult brain gene expression data and partial least squares analysis to find the weighted gene expression pattern that was most colocated with the cortical map of schizotypy-related magnetization.

**Results:**

Magnetization was significantly correlated with schizotypy in the bilateral posterior cingulate cortex and precuneus (and for disorganized schizotypy, also in medial prefrontal cortex; all false discovery rate–corrected *p*s *<* .05), which are regions of the default mode network specialized for social and memory functions. The genes most positively weighted on the whole-genome expression map colocated with schizotypy-related magnetization were enriched for genes that were significantly downregulated in two prior case-control histological studies of brain gene expression in schizophrenia. Conversely, the most negatively weighted genes were enriched for genes that were transcriptionally upregulated in schizophrenia. Positively weighted (downregulated) genes were enriched for neuronal, specifically interneuronal, affiliations and coded a network of proteins comprising a few highly interactive “hubs” such as parvalbumin and calmodulin.

**Conclusions:**

Microstructural magnetic resonance imaging maps of intracortical magnetization can be linked to both the behavioral traits of schizotypy and prior histological data on dysregulated gene expression in schizophrenia.

SEE COMMENTARY ON PAGE 212

The genetic architecture of schizophrenia spectrum disorders assumes many independent allelic variations, each of small effect, contributing to the probability of diagnosis. Individuals with the greatest accumulation of genetic risk have the more severe psychotic disorder; individuals with a lower genetic risk may have less severe, nonpsychotic schizotypal personality disorder (1), characterized by social eccentricity and unusual beliefs (2). The genetic risk for schizophrenia has been resolved more clearly by recent genome-wide association studies (3,4) and postmortem human brain transcriptional studies (3,5). However, it remains unclear how expression of these schizophrenia-related genes might be related to neuroimaging markers of schizophrenia spectrum disorders.

Macrostructural magnetic resonance imaging (MRI) studies—which measure anatomical parameters like cortical thickness—have collectively provided robust evidence for reduced volume or thickness in a network of interconnected cortical areas in patients with schizophrenia (6). There have been fewer MRI studies of schizotypy, and the pattern of macrostructural results has not been consistent, perhaps reflecting their relatively small sample sizes (Table 1).

**Table 1:** Previous Studies Relating Schizotypal Traits and Macrostructural Magnetic Resonance Imaging Metrics

Study	Sample Size, *n*	Mean Age, Years	Experimental Design	Brain Coverage	Schizotypy Measure	Type I Error Control	Structural Index	Directionality	Brain Regions
Evans *et al.*, 2016 (63)	28	11	VBM	Whole brain	PSI-C	Bonferroni	Volume	−	Caudate, amygdala, hippocampal gyrus, middle temporal
Nenadic *et al.*, 2015 (64)	59	31	VBM / positive and negative factor of schizotypy	Whole brain	CAPE	FWE	Volume	−	R precuneus
Wang *et al.*, 2015 (65)	69	19	VBM / dividing subjects with high/low schizotypy	Whole brain	SPQ	AlphaSim permutation	Volume	−	Dorsolateral prefrontal cortex, insula, posterior temporal, cerebellum
DeRosse *et al.*, 2015 (66)	138	36	ANCOVA / dividing subjects with high/low schizotypy	ROIs	SPQ	None	Thickness/GM and WM volume	−	Frontal, temporal
Kühn *et al.*, 2012 (67)	34	36	Vertexwise / positive and negative factor of schizotypy	Whole cortex/thalamus	SPQ	FDR	Thickness/thalamus volume	+	R dorsolateral prefrontal cortex, R dorsal premotor
Ettinger *et al.*, 2012 (68)	55	27	VBM	Whole brain	RISC	FWE cluster	Volume	−	Medial prefrontal, orbitofrontal, temporal
Modinos *et al.*, 2010 (69)	38	20	VBM / dividing subjects with high/low schizotypy	Whole brain	CAPE	FDR	Volume	+	Medial posterior cingulate, precuneus
Moorhead *et al.*, 2009 (70)	98	16	VBM / longitudinal study	Whole brain	SIS	Unspecified multiple comparison correction	Volume	−	L medial temporal, L amygdala, L parahippocampal gyrus
Stanfield *et al.*, 2008 (71)	143	16	ANCOVA	Whole brain	SIS	None	Folding	+	R prefrontal

Directionality refers to whether the study reports a positive (+) or negative (−) association between a schizotypy measure and a structural metric.

ANCOVA, analysis of covariance; CAPE, Community Assessment of Psychic Experience; FDR, false discovery rate; FWE, familywise error; GM, gray matter; L, left; PSI-C: Psychiatric and Schizotypal Inventory for Children; R, right; RISC, Rust Inventory of Schizotypal Cognitions; ROI, region of interest; SIS, Structured Interview for Schizotypy; SPQ, Schizotypal Personality Questionnaire; VBM, voxel-based morphometry; WM, white matter.

Microstructural MRI provides information about the composition of tissue within a voxel (7). For example, magnetization transfer (MT) images (8) and “myelin maps” derived from the ratio of conventional T1- and T2-weighted images (9) are sensitive to the proportion of fatty brain tissue represented by each voxel, which, according to histological studies on animal models, is related to myelin content (10, 11, 12, 13). MT maps have been used as markers of myelination in white matter and the cortex (14) in healthy subjects (15) and in demyelinating disorders such as multiple sclerosis (16, 17, 18). Schizophrenia has been associated with reduced MT in the frontal, temporal, and insular cortices (19, 20, 21, 22, 23, 24, 25, 26), and the cortical expression of schizophrenia-related genes was (negatively) correlated with T1- and T2-weighted maps (27).

In this context, we measured schizotypy, using the Schizotypal Personality Questionnaire (SPQ), and MT, using a multiparameter MRI scanning procedure, in a sample of 248 healthy young people (14–25 years of age) (Table S1). We tested 3 key hypotheses in a logical sequence: 1) that intracortical MT was correlated with the SPQ total score (and subscale scores), 2) that the cortical pattern of schizotypy-related magnetization (SRM) was colocated with a cortical map of weighted whole-genome expression, and 3) that the gene transcripts most strongly coupled to SRM were enriched for genes that were transcriptionally dysregulated in histological case-control studies of schizophrenia.

## Methods and Materials

### Participants

A total of 2135 healthy young people, 14 to 25 years of age, were recruited from schools, colleges, National Health Service primary care services, and direct advertisement in north London and Cambridgeshire, United Kingdom. This primary cohort was stratified into 5 contiguous age-related strata, balanced for gender and ethnicity (28). A secondary cohort of 297 individuals was recruited by randomly subsampling the primary cohort so that ∼60 participants were assigned to each of the same age-related strata, balanced for gender and ethnicity, as in the primary cohort. Participants were excluded if they had a current or past history of clinical treatment for a psychiatric disorder, drug or alcohol dependence, neurological disorder including epilepsy, head injury causing loss of consciousness, or learning disability (see Supplement for details).

Written informed consent was provided by all participants as well as written parental assent for participants less than 16 years of age. The study was approved by the National Research Ethics Service and conducted in accordance with National Health Service research governance standards.

### Schizotypy Assessment

The SPQ (29) is a self-report scale, comprising 74 dichotomous items that are grouped on 9 subscales, measuring the complex trait of schizotypy. Participants completed the SPQ on 2 assessments, separated by 6 to 18 months, so that traitlike scores on total and subscale SPQ metrics could be estimated by the number of questionnaire items positively endorsed by each participant on average over time.

### MRI Data Acquisition

Structural MRI scans were acquired on 1 of 3 identical 3T MRI systems in London or Cambridge, United Kingdom (Magnetom TIM Trio [Siemens Healthcare, Erlangen, Germany], software version VB17). The multiparametric mapping protocol (8) yielded 3 multiecho fast low-angle shot scans with variable excitation flip angles. By appropriate choice of repetition time and flip angle α, acquisitions were predominantly weighted by T1 (repetition time = 18.7 ms, α = 20°), proton density, or MT (repetition time = 23.7 ms, α = 6°). Other acquisition parameters were 1-mm^3^ voxel resolution, 176 sagittal slices, and a field of view of 256 × 240 mm. A pilot study demonstrated satisfactory levels of between-site reliability in multiparametric mapping data acquisition (8). MT images (15) and T1 images (30,31) from this sample have been previously reported.

### MRI Reconstruction, Cortical Parcellation, and Estimation of SRM

We used a standard automated processing pipeline for skull stripping, tissue classiﬁcation, surface extraction, and cortical parcellation (FreeSurfer [http://surfer.nmr.mgh.harvard.edu]) applied to longitudinal relaxation rate (R1) maps (R1 = 1/T1). Expert visual quality control ensured accurate segmentation of pial and gray/white matter boundaries. Regional MT values were estimated at each of 68 cortical regions for each subject, resulting in a 248 × 68 regional MT data matrix. The Euler number for the R1 images was calculated as a proxy measure of image quality in the simultaneously acquired MT images (32).

A simple linear model of age-related change in MT was used to estimate two key parameters for each region: baseline MT at 14 years of age and age-related rate of change in the period from 14 to 24 years of age (15). For the principal analyses, effects of age on MT were controlled by regression before estimating the correlation of the age-corrected MT residuals with SPQ total score. The Kolmogorov-Smirnov normality test was used to determine the appropriate correlation estimator (Pearson’s or Spearman’s).

### Estimation of Regional Gene Expression

We used the Allen Human Brain Atlas (AHBA), a whole-genome expression atlas of the adult human brain created by the Allen Institute for Brain Sciences using 6 donors 24 to 57 years of age (http://human.brain-map.org) (33). Probe-to-gene and sample-to-region mapping strategies can have a major impact on regional gene expression estimation (34). Here, we used the genome assembly hg19 (http://sourceforge.net/projects/reannotator/) (35) to reannotate the probe sequences into genes (36). When genes were mapped by multiple complementary RNA hybridization probes, the probe showing the highest average expression across samples was selected (37). MRI images of the AHBA donors were parcellated using the Desikan-Killiany atlas, and each cortical tissue sample was assigned to an anatomical structure. Regional expression levels were compiled to form a 68 × 20,647 regional transcription matrix (38) (see Supplement).

### SRM and Human Brain Gene Expression

We used partial least squares (PLS) to analyze covariation between SRM and gene expression because it is technically well suited to the high collinearity of the gene expression data (39,40), and because PLS and the related multivariate method of canonical correlation analysis have been extensively developed and used for neuroimaging and transcriptional data analysis (41, 42, 43, 44). Specifically, we used PLS to analyze the relationship between the vector of 68 regional measures of SRM and the 68 × 20,647 matrix of 68 regional messenger RNA measurements for 20,647 genes (44). The first PLS component (PLS1) was defined as the weighted sum of whole-genome expression that was most strongly correlated, or most closely colocated, with the anatomical map of SRM. Permutation testing based on spherical rotations or “spins” of the spatially correlated SRM map was used to test the null hypothesis that PLS1 explained no more covariance between SRM and whole-genome expression than expected by chance (31). Bootstrapping was used to estimate the variability of each gene’s positive or negative weight on PLS1, and we tested the null hypothesis of zero weight for each gene with a false discovery rate (FDR) of 5% (42). The set of genes that were significantly (positively or negatively) weighted on PLS1 was called the SRM gene list or set.

### Enrichment Analysis

We assigned a cellular affiliation score to each gene in the SRM gene list according to prior criteria for 4 cell types—neuron, astrocyte, microglia, or oligodendroglia (45)—and for a more fine-grained set of cell types (46) (Table S2). We used a data resampling procedure to test the null hypothesis that SRM genes were randomly assigned to different cell types.

We used 2 lists of genes that were differentially expressed, or transcriptionally dysregulated, in postmortem brain tissue measurements of messenger RNA from case-control studies of schizophrenia: 1) the list of genes reported by Gandal *et al.* (5) as upregulated (*n* = 845) or downregulated (*n* = 1175) in the prefrontal and parietal brain regions in schizophrenia (*n* = 159); and 2) the list of genes reported by Fromer *et al.* (47) as upregulated (*n* = 304) or downregulated (*n* = 345) in the dorsolateral prefrontal cortex in schizophrenia (*n* = 258). The 2 gene lists were partially overlapping (Table S3), and differential expression of all genes subsumed by the union of the 2 lists was strongly correlated between studies (ρ = .76, *p* < 10^−129^) (Figure S1).

We used repeated random relabeling of genes to test the null hypothesis that the SRM gene list included no more schizophrenia-related genes than expected by chance. We also applied the same resampling procedures to comparable prior data on differential gene expression from case-control studies of inflammatory bowel disease, bipolar disorder (BPD), major depressive disorder, and autism spectrum disorder (ASD) (5).

### Data and Code Sharing

#### Data

Regional MT for 68 cortical regions, schizotypy scores, age, gender, socioeconomic status, scanning site, total brain volume, and Euler values (for *N* = 248) are available at https://github.com/RafaelRomeroGarcia/Schizotypy_MT_geneExp.

#### Code

Cortical parcellation of gene expression maps to estimate regional mean gene expression (48) can be found at https://github.com/RafaelRomeroGarcia/geneExpression_Repository. The rotate_parcellation code generates null models that preserve the spatial contiguity of cortical regions for permutation testing (31): https://github.com/frantisekvasa/rotate_parcellation. PLS analysis and bootstrapping to estimate PLS weights (15) can be found at https://github.com/KirstieJane/NSPN_WhitakerVertes_PNAS2016/tree/master/SCRIPTS. To generate Figure S1 from the raw Gandal *et al.* (5) and Fromer *et al.* (47) datasets, we used https://github.com/RafaelRomeroGarcia/Schizotypy_MT_geneExp. For mapping regional values to the cortical surface for visualization (BrainsForPublication v0.2.1), see https://doi.org/10.5281/zenodo.1069156.

## Results

### Sample Characteristics

After quality control checks, complete, evaluable MRI and behavioral data were available for analysis on 248 participants: mean age 19.11 ± 2.93 years; 123 (50%) female participants; 213 (86%) right-handed participants; IQ = 112.0 ± 10.5; and 214 (86%) white Caucasian, 10 Asian, 4 black/African American/Caribbean American, 17 mixed, and 3 other ethnic groups (see Table S1 for details).

### Schizotypy and MT

Schizotypal personality scores in this healthy (nonpsychotic) sample followed a positively skewed distribution (mean = 0.23, median = 0.20) that was normalized by square root transformation prior to statistical analysis. There was no significant effect of age (*R*^2^ < .001, *p* = .69) (Figure S2), gender (*R*^2^ < .001, *p* = .77), or age-by-gender interaction (*R*^2^ < .001; *p* = .82) on SPQ total score or subscale scores.

**Figure 1:**
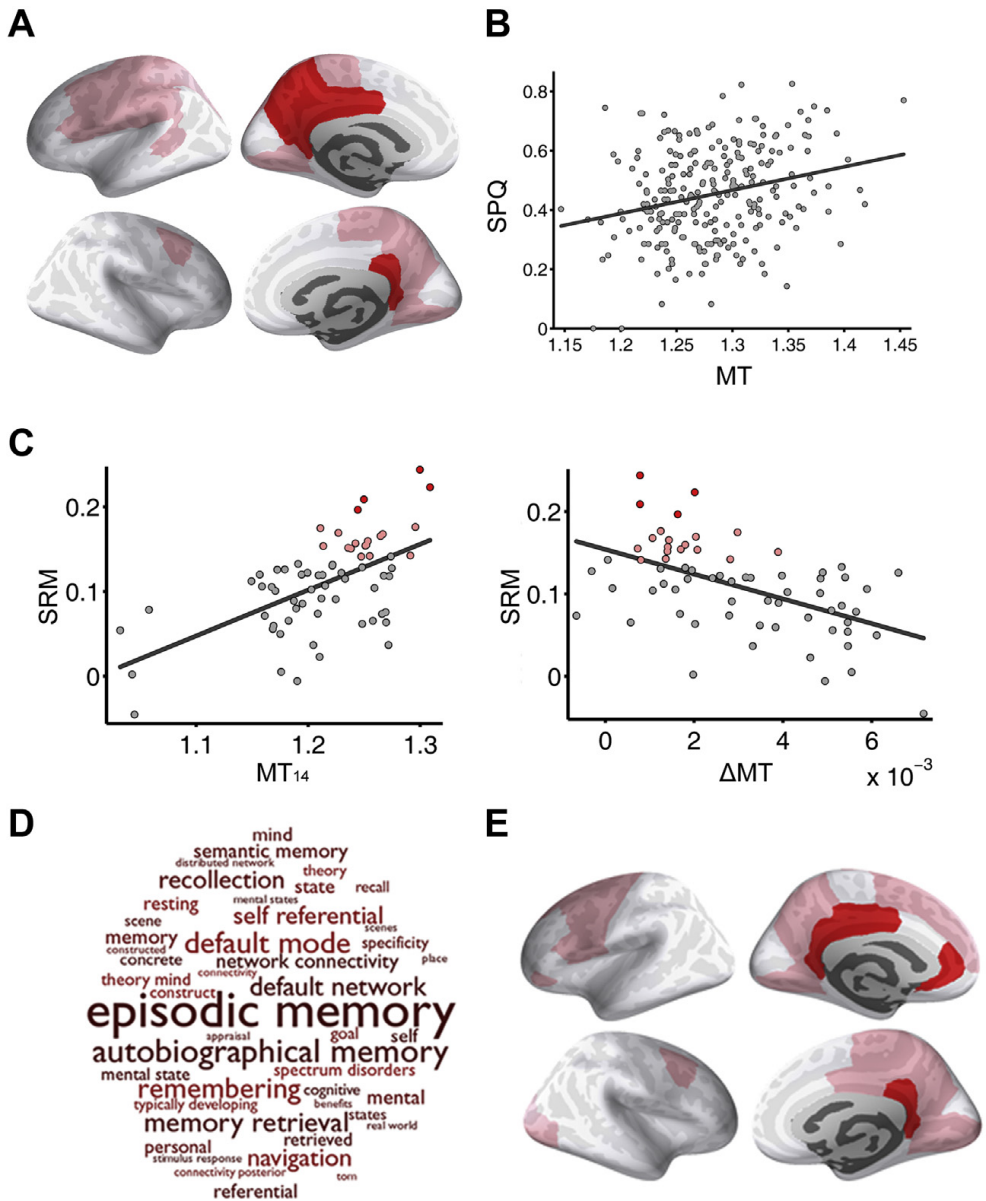
Schizotypy-related magnetization (SRM): association between intracortical magnetization transfer (MT) and Schizotypal Personality Questionnaire (SPQ) score. **(A)** Cortical surface maps highlighting areas where SPQ total score was significantly positively correlated with regional MT after controlling for age by regression: pink regions had nominally significant SRM (2-tailed *p <* .05); red regions had significant SRM controlled for multiple comparisons over 68 cortical regions tested (false discovery rate < .05). **(B)** Scatterplot of SPQ total score for each participant vs. mean MT in regions of significant SRM (*R*^2^_246_ = .04, *p* = .002); each dot represents 1 of 248 healthy people 14 to 25 years of age. **(C)** Scatterplots of SRM vs. MT at 14 years of age (MT_14_) (left) (*R*^2^_67_ = .34, permutation testing based on spherical rotations: *p* = .002) and SRM vs. change in magnetization 14 to 25 years of age (ΔMT) (right) (*R*^2^_67_ = .28, permutation testing based on spherical rotations: *p* = .006). Each point represents a cortical region and colored points represent regions with significant SRM (pink: *p <* .05; red: false discovery rate < .05). **(D)** Word cloud representing ontological terms most frequently associated with functional activation of the medial posterior cortical areas of significant SRM. **(E)** Cortical surface maps highlighting areas where scores on the disorganized factor of schizotypy was significantly positively correlated with regional MT after controlling for age by regression (pink, 2-tailed *p <* .05; red, false discovery rate < .05).

SPQ total score was modestly positively correlated with global MT, estimated as the average MT over all 68 regions (*R*^2^ = .02, *p =* .015) (Figure S3). SPQ total score was significantly correlated with age-corrected regional MT in 4 of 68 regions individually tested (*R*^2^ > .04, *p <* .05, FDR corrected) (Figure 1A, B and Table S4): the left isthmus cingulate, left posterior cingulate, left precuneus, and right isthmus cingulate. These medial posterior cortical regions had high MT signals at 14 years of age (MT_14_) and relatively slow rates of increase in MT over the period of 14 to 25 years of age (ΔMT) (Figure 1C).

We used prior functional MRI data to identify experimental task conditions that were most robustly associated with functional activation of these areas of significant SRM (Neurosynth [https://neurosynth.org/]) (49): memory, social cognition or theory of mind, and executive functions (Figure 1D). The posterior cingulate and medial parietal cortical areas of significant SRM were also enriched for default mode network–related terms in ontological analysis of prior functional MRI data (49).

### Sensitivity Analyses of SRM

We used a linear model to control the association between SPQ and MT for the potentially confounding effects of age, gender, site, socioeconomic status, and total brain volume (Figure S4), and robust estimators to mitigate the influence of the small number of high SPQ scores on the estimation of SRM (Figure S5). In both cases, the key results were conserved: namely, significant SRM in default mode network areas and significant correlations between PLS1 weights and differential gene expression in schizophrenia. We also noted a negative correlation between Euler number and global MT, indicating reduced MT in a minority of poor-quality images (ρ = −.14, *p =* .03). When we excluded the 10% of participants with the poorest image quality, the correlation between Euler number and MT was no longer significant (ρ = .04, *p =* .11), but the key results were conserved (Figure S6).

Schizotypy is a complex trait comprising multiple dimensions of cognition, emotion, and behavior. In addition to the principal analysis of SPQ total score, we also considered 2 possible decompositions of the schizotypal trait. Nine subscales of the SPQ defined by Raine (29) were positively correlated with regional MT, but these associations were less robust than for SPQ total score (Figure S7). All 3 factors of schizotypy defined by Raine *et al.* (50), i.e., positive, negative, and disorganized dimensions, were positively correlated with MT. The correlation between disorganized schizotypy and MT was strongest and statistically significant when controlling for multiple comparisons (Figure 1E and Figure S8).

We also measured cortical thickness for each of the same 68 regions, using R1 images collected as part of the same MRI sequence used to measure MT. SPQ scores were negatively correlated with cortical thickness in some regions, but the associations between schizotypy and cortical thickness were not significant when corrected for multiple tests (Figure S9).

### SRM and Gene Expression

**Figure 2:**
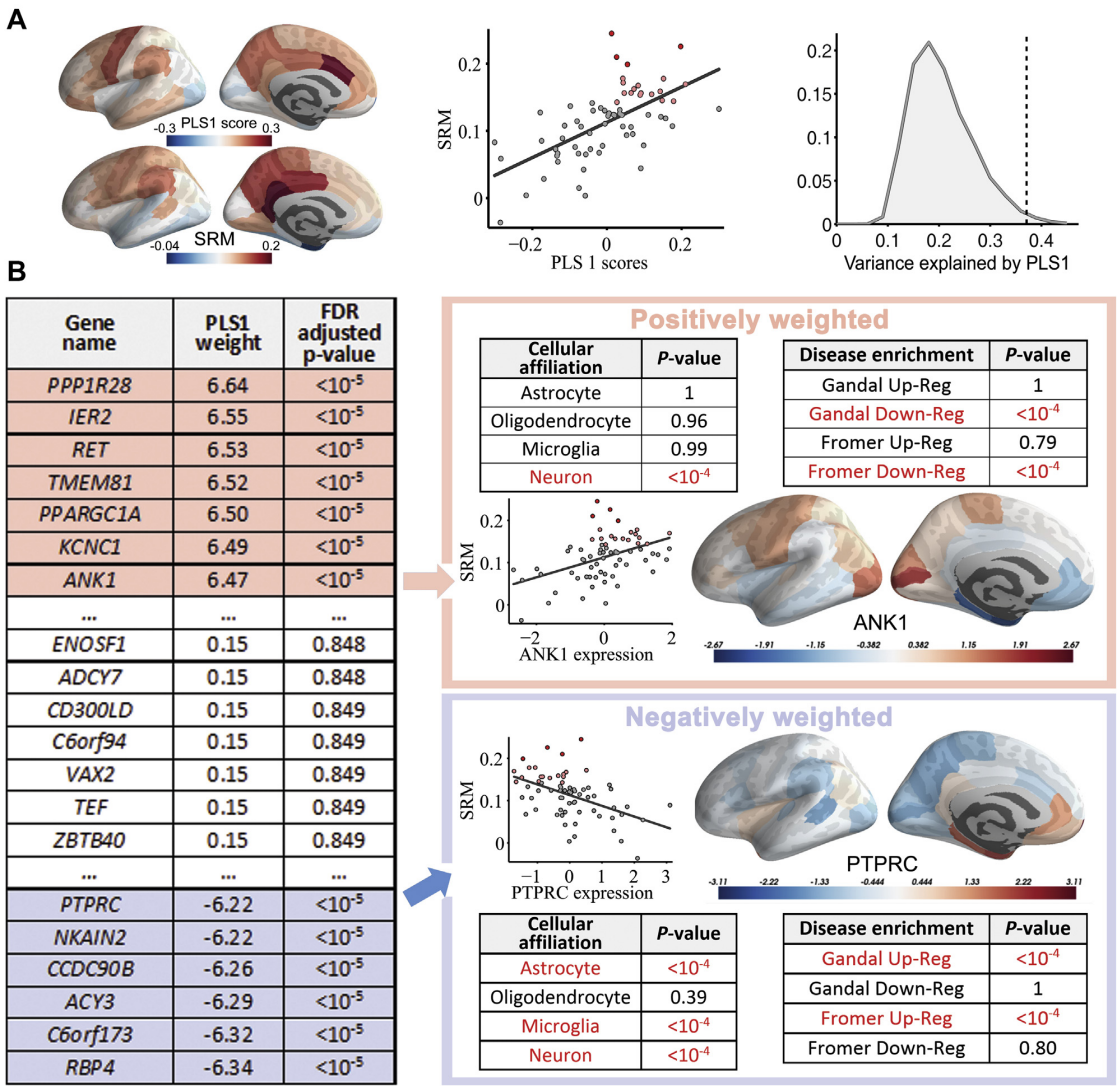
Gene expression and schizotypy-related magnetization (SRM). **(A)** (Left panel) The first partial least squares component (PLS1) defined a linear combination of genes that had a similar cortical pattern of expression to the cortical map of SRM, representing the correlation between Schizotypal Personality Questionnaire and magnetization transfer at each of 68 cortical regions. (Center panel) Scatterplot of PLS1 scores versus SRM; each point is a cortical region. (Right panel) The combination of genes defined by PLS1 explains more of the variance in SRM (dotted line) than expected by chance (histogram of permutation distribution). **(B)** Illustrative example of the weights assigned to representative genes on PLS1. Genes with the highest positive weights are colored in pink, nonsignificantly weighted genes are shown in white, and the genes with the lowest negative weights are colored in blue. Tables summarize *p* values by permutation testing for enrichment analysis by 4 lists of genes affiliated to specific cell types and 4 lists of genes associated with schizophrenia: Gandal and Fromer up-reg (5, 47) are lists of genes transcriptionally upregulated postmortem in schizophrenia; Gandal and Fromer down-reg are lists of genes transcriptionally downregulated in schizophrenia. Scatterplots and cortical maps illustrate that positively weighted genes, like *ANK1*, are overexpressed in cortical regions with high levels of schizotypy-related myelination, whereas negatively weighted genes, like *PTPRC*, are underexpressed in regions with high levels of SRM. FDR, false discovery rate.

PLS1 defined a weighted sum of whole-genome expression that accounted for ∼40% of the cortical patterning of SRM, significantly more than expected by chance (permutation testing based on spherical rotations: *p* = .027) (Figure 2A).

Multiple univariate *Z* tests were used to test the set of null hypotheses that the weight of each gene on PLS1 was zero. We found that this null hypothesis was refuted for 1932 positively weighted genes and 2153 negatively weighted genes (*p <* .05, FDR corrected for whole-genome testing at 20,647 genes) (Figure 2B). Positively weighted genes were normally overexpressed, and negatively weighted genes were normally underexpressed, in cortical areas with high SRM. These 4085 genes constituted the SRM gene list.

### Functional and Schizophrenia-Related Enrichment of SRM Genes

The SRM gene list was tested for enrichment by genes characteristic of specific cell types using 2 sets of prior criteria (45,46). Positively weighted SRM genes were enriched for neuronal affiliation (permutation test, *p* < 10^−4^) (45) and, more specifically, for genes differentially expressed in fast-spiking parvalbumin (*PVALB*)-positive inhibitory interneurons (permutation test, FDR-corrected *p <* .01) (Table S2) (46). Negatively weighted SRM genes were enriched for astrocytes, microglia, and neuronal affiliation (permutation tests, all *p*s < 10^−4^) (Figure 2B) (45,46).

The positive or negative weighting of each SRM gene was strongly related to its differential expression in 2 postmortem studies of schizophrenia (5,47). Positively weighted SRM genes were enriched for genes that were significantly downregulated in both studies (Table S5) but not for significantly upregulated genes in either study. Additionally, positively weighted SRM genes were also enriched for genes previously associated with white matter connectivity differences in schizophrenia described by Romme *et al.* (51) (Figure S10). In contrast, negatively weighted SRM genes were enriched for genes that were significantly upregulated in both studies (permutation tests, all *p*s < 10^−4^) (Figure 2B and Table S6) but not for significantly downregulated genes.

**Figure 3:**
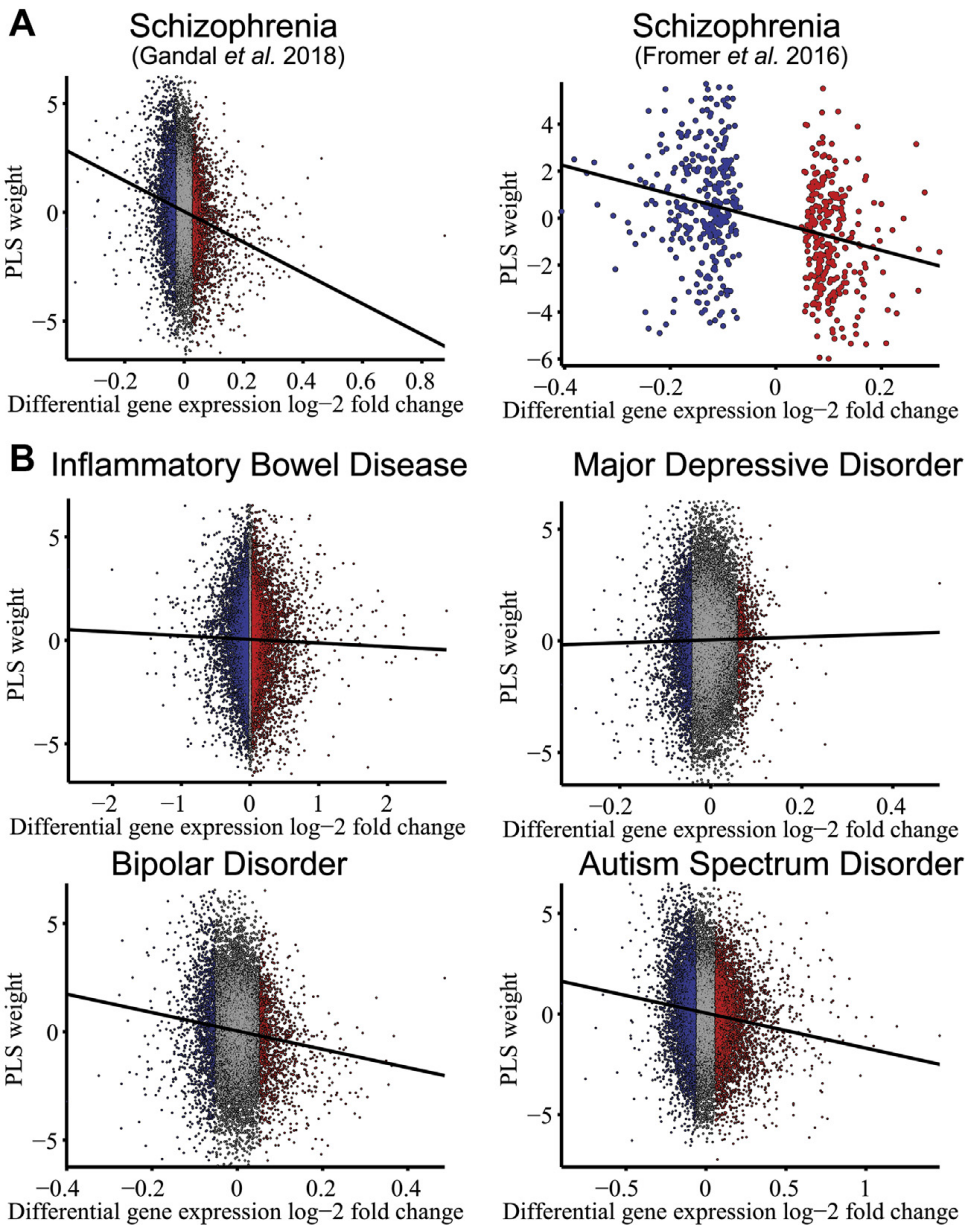
Weights of gene expression from partial least squares (PLS) analysis of schizotypy-related magnetization (SRM) were related to histological measures of differential gene expression from case-control studies of schizophrenia and other disorders. **(A)** The weight of each gene on the first PLS component was significantly negatively correlated with differential gene expression postmortem in schizophrenia according to prior data reported by Gandal *et al.* (5) (Spearman’s rank correlation, ρ_11111_ = −.16, Bonferroni-corrected adjusted *p*_11111_ < 10^−65^) and by Fromer *et al.* (47) [Spearman’s rank correlation, ρ_586_ = −.30, adjusted *p*_586_ < 10^−12^; for this dataset, only significantly different expression values have been reported (46)]. **(B)** Correlations between PLS weights and differential expression were also evaluated for other conditions (5): inflammatory bowel disease (ρ_586_ = −.02, adjusted *p*_586_ = .10), major depressive disorder (ρ_15281_ = .007, adjusted *p*_15281_ = .37), bipolar disorder (ρ_16064_ = −.09, adjusted *p*_16064_ < 10^−19^ ), and autism spectrum disorder (ρ_11131_ = .11, adjusted *p*_11131_ < 10^−35^). Red and blue points represent genes that are significantly up- and downregulated in postmortem data.

Convergently, there was a significant negative correlation (Spearman’s ρ = −.16, *p* < 10^−6^) between the PLS1 weights of all genes in the genome and the differential expression values reported for all genes by Gandal *et al.* (5) and Fromer *et al.* (47) (Figure 3A). PLS1 gene weights were not correlated with differential expression in inflammatory bowel disease or major depressive disorder; however, they were negatively correlated with differential expression in BPD and ASD (Figure 3B).

**Figure 4:**
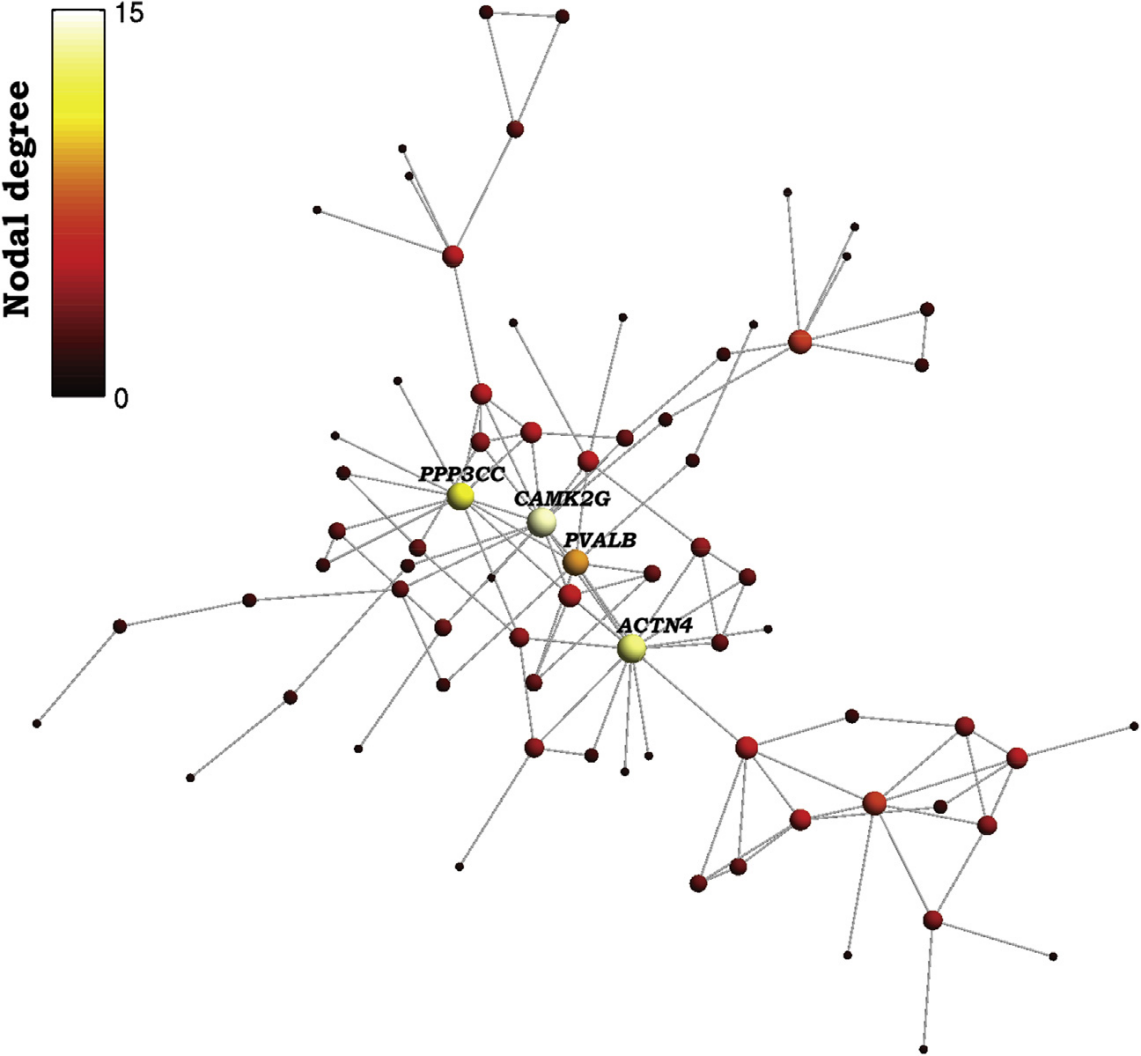
Protein-protein interaction network for a set of 213 proteins coded by genes associated with both schizotypy-related magnetization and postmortem brain transcriptional dysregulation in schizophrenia. Nodes represent genes that were both 1) downregulated in brain tissue from 159 patients with schizophrenia and 2) positively weighted on the partial least squares component most strongly associated with schizotypy-related myelination in 248 healthy adolescents. Edges represent known protein-protein interactions. The color and size of each node represents its degree centrality or “hubness,” simply the number of interactions that protein has with the other proteins in the network. The top 4 most highly connected hubs are highlighted: *PPP3CC* is a calmodulin dependent phosphatase, calcineurin; *CAMK2G* is a calcium/calmodulin dependent kinase; *PVALB* is a calcium binding protein, parvalbumin; *ACTN4* is a microfilamentous protein, actinin-alpha-4. This network is specialized for calcium-dependent processes that have been previously associated with interneurons and with pathogenesis of schizophrenia. For the complete list of gene names on the protein-protein interaction network, see Figure S11 and see https://version-10-5.string-db.org/cgi/network.pl?taskId=RMpA04wbWG8k for a full interactive version of the protein-protein interaction network.

We analyzed the network of known protein-protein interactions [STRING (http://string-db.org) (52)] between proteins coded by the 213 genes that were significantly downregulated in schizophrenia (5) and significantly positively weighted in the PLS analysis of SRM (Table S5). There were significantly more interactions (edges) between proteins coded by these genes than expected by chance (permutation test, *p* < 10^−5^) (Figure 4 and Figure S11). Topologically, the network comprised several clusters of densely interconnected and functionally specialized proteins. The biggest cluster was enriched for synaptic terms and centered around highly connected “hub” proteins (Figure 4).

## Discussion

Schizotypy was associated with intracortical magnetization of posterior cortical regions of the default mode network and colocated with a normative cortical pattern of weighted whole-genome expression. The gene transcripts most strongly weighted in association with this MRI map of SRM were significantly enriched for genes specifically expressed by neuronal and glial cells, especially *PVALB*-positive interneurons, and for genes that were transcriptionally dysregulated in 2 prior postmortem studies of schizophrenia.

### Magnetization, Intracortical Myelination, and Schizotypy

MT is a microstructural MRI measurement that is sensitive to the ratio of fatty and watery tissue represented by each voxel, and in the brain, most of the fat is myelin. Intracortical myelination, especially of the deeper layers of the cortex, has been recognized since seminal cytoarchitectonic and myeloarchitectonic studies in the early 20th century (14). MT images represent a stark contrast between the cortex and the central white matter as well as more nuanced variations across different cortical areas and layers (53). Histological measurements of myelin were positively correlated with MRI measurements of MT in postmortem brains (54). Intracortical measurements of MT in humans have been validated as microstructural MRI markers of myelination in healthy volunteers (55) and in patients with multiple sclerosis (56).

One plausible interpretation of SRM, therefore, is as a proxy marker for a biological state of schizotypy-related myelination. On this assumption, the results are open to further interpretation at a cellular level. For example, greater “myelination” could imply a greater density of myelinated neurons per voxel (a neuronal process), or a greater density of myelin per neuron (an oligodendroglial process), or some combination of these and other cellular parameters. The data available to us did not allow direct resolution of the relationships between SRM and myelination. Instead, we used open data on human brain gene expression (*n* = 6, mean age = 42.5 years) to explore these questions more indirectly.

The adult brain gene expression profile that was most closely colocated with the adolescent brain map of SRM (*n* = 248, mean age = 19 years) was enriched for neuronal, but not oligodendroglial, affiliations. This pattern of results arguably favors the interpretation that higher MT indicates a greater density of myelinated neurons per voxel, rather than a greater density of myelin per neuron, in people with higher schizotypy scores. However, the 20+-year age gap between the MRI measurements and the messenger RNA measurements precludes definitive resolution of these and other possible cellular interpretations of SRM. Although the SRM gene set is not known to demonstrate major developmental changes in expression after childhood (Figure S12), in the future it will be important to colocate MT phenotypes in children and young people (and animal models) with more precisely age-matched data on brain gene expression and histology.

The macroscopic medial posterior cortical areas where MT was most strongly correlated with schizotypy in general—SPQ total score—are key components of the default mode network [as defined by functional MRI studies (57)] and specialized for memory, social cognitive, and theory-of-mind functions that are known to be abnormal in patients with schizophrenia (58). Interestingly, the schizotypal factor of disorganization was also correlated with MT in the medial prefrontal cortical areas that also form part of the default mode network. All these regions had high levels of magnetization at 14 years of age and no significant subsequent change in magnetization over the period 14 to 25 years of age. This contrasts with areas of the lateral association cortex, which have a low level of MT at 14 years of age but show significant increase in MT over the course of adolescence (15). We can infer that these medial posterior cortical areas matured as part of a preadolescent wave of cortical development (59), which would be compatible with the stable, traitlike properties of schizotypal personality in these data and in other studies of adolescents and adults.

### Cortical Gene Expression, Schizotypy-Related Myelination, and Schizophrenia

We wanted to identify which genes in the whole genome had a cortical expression pattern that was most similar to the cortical map of SRM. A large number (>20,000) of nonindependent statistical tests would be entailed in testing the association between each transcript’s spatially correlated cortical expression map and the cortical map of SRM. Therefore, we favored a multivariate approach and used PLS to identify a cortical pattern of weighted whole-genome expression that was significantly colocated with the SRM map and to identify which particular gene transcripts were most positively or negatively weighted.

We found that the positively weighted genes (*n =* 1932) were overexpressed in cortical areas with high levels of SRM, whereas the negatively weighted genes (*n =* 2153) were overexpressed in cortical areas with low levels of SRM. Both positive and negative genes were enriched for neuronal, but not for oligodendroglial, affiliations. Positive genes were specifically enriched for *PVALB*-positive inhibitory interneurons; negative genes were enriched for astrocytes and microglia.

We predicted hypothetically that the SRM gene set would be enriched for genes that are known to be transcriptionally dysregulated in schizophrenia. This prediction was supported by convergent results from enrichment analysis using two prior, independently discovered, and partially overlapping lists of genes differentially expressed in postmortem case-control studies of schizophrenia. In both cases, there were significantly more histologically downregulated genes in the list of positively weighted SRM genes, and significantly more upregulated genes in the list of negatively weighted SRM genes, than expected by chance. In other words, genes with reduced brain transcription postmortem in schizophrenia were normally more highly expressed in cortical areas with higher levels of SRM. A subset of the positively weighted SRM genes has been previously associated with white matter dysconnectivity in schizophrenia (51), suggesting that SRM of cortex and schizophrenia-related disruption of central white matter tracts may be different imaging phenotypes related to expression of genes in common.

The subset of 213 SRM-positive genes that were also significantly downregulated in one or both of the prior histological studies coded for a protein-protein interaction network comprising a small number of highly connected hub proteins (*ACTN4* [alpha-actinin-4], *CAMK2G* [calcium/calmodulin-dependent protein kinase II gamma], *PPP3CC* [protein phosphatase 3 catalytic subunit gamma], and *PVALB*), each hub having up to 14 known biochemical interactions with other proteins in the network. *CAMK2G* is one of a family of serine/threonine kinases that mediate many of the second messenger effects of Ca^2+^ that are crucial for plasticity at glutamatergic synapses. *PVALB* is a calcium-binding albumin protein that is expressed particularly by the fast-spiking class of GABAergic (gamma-aminobutyric acidergic) interneurons that has been strongly implicated in the pathogenesis of schizophrenia (Figure 4) (60).

We found that the SRM gene list was also enriched by genes differentially expressed in ASD and BPD. These results are consistent with the postmortem evidence (5) that the differential gene expression profile of schizophrenia (compared with healthy controls) is strongly correlated with the case-control differences of transcription in BPD and ASD (ρ > .45, *p <* .001). They are also consistent with clinical evidence that both ASD and BPD are associated with increased schizotypal traits (61,62).

### Methodological Issues

The brain tissue samples used for RNA sequencing in the AHBA were not homogeneously distributed across the cortex, so estimates of regional expression are based on different numbers of experimental measurements in each of the 68 regions. The case-control differences in frontal or parietal lobar gene transcription reported by Gandal *et al.* (5) and Fromer *et al.* (47), although based on a relatively large number of patients, are not as precisely localized or representative of the whole brain as the AHBA and MRI data. This study has a considerably larger sample size than any previously reported MRI study of schizotypy, and it is the first to evaluate a microstructural MRI marker, which was more strongly related to schizotypy than the more conventional macrostructural MRI marker of cortical thickness. Nonetheless, it is theoretically surprising that there was limited evidence for significant SRM of the frontal and lateral temporal cortex (although magnetization of the medial prefrontal cortex was significantly associated with the disorganized component of schizotypy) (Figure 1E), possibly reflecting limited statistical power. The SPQ is a self-report questionnaire measure of schizotypy; more refined and objective assessments of schizotypal traits would likely add value to future studies.

### Conclusions

Overall, these correlational results do not unambiguously resolve questions of causality, but they are consistent with the interpretation that SRM, putatively an imaging marker of intracortical density of myelinated neurons, represents cellular processes determined in part by transcription of genes related to schizophrenia and other neuropsychiatric disorders.

## Supplementary Material

Supplemental Material

Key Resources Table
