# Supplementary material for: Schizotypy-Related Magnetization of Cortex in Healthy Adolescence Is Colocated With Expression of Schizophrenia-Related Genes
Source: Biol Psychiatry. 2020 Aug 1;88(3):248–59. doi: 10.1016/j.biopsych.2019.12.005 (PMC7369635; doi:10.1016/j.biopsych.2019.12.005)
Supplement: Supplemental Material [file mmc1.pdf]

Schizotypy-Related Magnetization of Cortex in Healthy Adolescence Is Co-Located With Expression of Schizophrenia-Related Genes

Supplemental Information

Contents

Supplementary Methods 2
Sample 2
MRI reconstruction, cortical parcellation and structural feature extraction 2
Estimation of regional gene expression 3
Partial Least Squares analysis 4
Supplementary Results 5
Protein-protein interaction network enrichment 5
Replication using only left hemisphere data 5
Supplementary Figures 6
Supplementary Tables 18
Supplementary References 32

Supplementary Methods

Sample

All primary cohort participants satisfied the following eligibility criteria: aged between 14 and 24 years inclusive; able to understand written and spoken English; willing and able to give informed consent for recruitment into the study cohort and consent to be re-contacted directly for possible participation in future studies within the consortium. They were excluded if they were currently or had recently (within the last 12 months) participated in a clinical trial of an investigational medical product. Participants completed questionnaire measures of socio-demographic status, family and educational or occupational environments, and sub-clinical psychopathology. All participants in the secondary MRI cohort satisfied the following additional eligibility criteria: willing and able to give informed consent for participation in the study and to attend full-day assessments at either the University of Cambridge or University College London study sites; have normal or corrected-to-normal vision. Participants were excluded if they had a current or past history of clinical treatment for a psychiatric disorder or for drug or alcohol dependence; had a current or past history of neurological disorders or trauma including epilepsy, or head injury causing loss of consciousness; had a learning disability requiring specialist educational support and/or medical treatment; or had a safety contraindication on the MRI scanning checklist. Secondary cohort participants attended one of the study sites (University of Cambridge or University College London) for a day of clinical, cognitive and MRI assessments. Socio-economic status was derived from the neighbourhood poverty index (NPI), provided by the UK Office of National Statistics (1).

MRI reconstruction, cortical parcellation and structural feature extraction

Freesurfer (recon-all) reconstructions were created for each participant using the longitudinal relaxation rate (R1) maps (R1=1/T1). Cortical thickness (CT) measurements were estimated by reconstructing the pial surface and the boundary between grey matter and white matter (2, 3) and measuring the distance between these surfaces. For each subject, MT-weighted images were projected on the cortical surface of each Freesurfer reconstruction (mri_vol2surf). CT and MT values were averaged across all vertices included in each of the 68 cortical parcel defined in the Desikan-Killiany atlas (4). This process was repeated for each subject resulting in a (248 × 68) matrix of cortical MT and a (248 × 68) matrix of CT at each of 68 regions for each of 248 participants.

Meta-analysis of structural maps using Neurosynth

To identify the cognitive functions served by the medial posterior cortical regions showing a significant correlation between MT and SPQ, a meta-analysis was performed using the

Neurosynth software package (http://neurosynth.org; (5)). Neurosynth uses a combination of text mining, meta-analysis and machine-learning techniques to aggregate coactivation results from 11,406 functional MRI (fMRI) studies and links particular brain regions with particular cognitive functions based on keywords from the functional studies (5). This software was used here to find keywords associated with prior published findings correlated with the schizotypy-related myelination map. Neurosynth calculated the likelihood of each term given the activation described by the schizotypy-related myelination map -*i.e.* P(term | Activation map)-. The top 100 keywords (excluding anatomical terms for brain regions) ranked by the correlation strength (semantic association) between the MRI map and the meta-analytic map were visualized as a word cloud, with the size of the font scaled by correlation strength. This allowed us to identify the most likely reverse inferences that would be drawn if the schizotypy-related myelination map were a functional imaging map.

Estimation of regional gene expression

We used the Allen Human Brain Atlas (AHBA) – a whole-genome, whole-brain transcriptomic dataset of the adult human brain created by the Allen Institute for Brain Sciences (http://human.brain-map.org) (6). The AHBA includes six donors, three Caucasian, two African-American and one Hispanic. Their ages are 57, 55, 49, 39, 31 and 24 years. Further details on microarray analysis are available from the Allen Institute (www.brain-map.org). According to AHBA annotation, 48171 out of 58692 probes can be mapped onto genes. Here, we used Richiardi et al.'s (2015) criterion to reannotate the probe sequences using the genome assembly hg19 (UCSC Genome Browser; http://sourceforge.net/projects/reannotator/; (7)). After the reannotation, a final set of 49770 probes was associated with genes. Previous studies have shown the high between-study consistency of this probe-to-gene mapping method (8). Generally, multiple cRNA hybridization probes were used for each gene and, in these cases, we selected the probe with the highest mean gene expression. This is in line with recommendations from a recent study evaluating the impact of various preprocessing decisions on the correspondence between microarray and RNA-seq expression (9). Probes that were not matched to gene symbols in the AHBA data were excluded, resulting in 20,467 gene expression values that were evaluated in 3,702 brain samples. Due to the similarity of gene expression between hemispheres, the AHBA only sampled two of the four donors in the right hemisphere. In order to increase the number of AHBA samples per cortical region, all samples were pooled between bilaterally homologous cortical areas.

The anatomical structure associated with each tissue sample was determined using the MRI data provided by the AHBA for each donor. T1-images of the six donors were processed

following the Freesurfer pipeline. Briefly, cortical surface reconstruction of each donor performed by Freesurfer recon-all involves (*i*) non-uniformity intensity correction, (*ii*) resampling to isotropic images of 1mm, *(iii)* registration to Talairach space, (*iv*) an initial WM segmentation that is used to generate a tessellated representation of the WM/GM boundary, (*v*) deformation of the tessellated WM/GM boundary outwards to the volume that maximizes the CSF/GM contrast to generate the pial surface and (*v*i) automatic correction of geometrical topological abnormalities. The Desikan-Killiany atlas employed in the neuroimaging dataset was reconstructed in each AHBA donor brain. Surface-based parcellation of each donor's brain was transformed into a volumetric parcellation that covered the whole cortical mantle. 73% of the total AHBA cortical samples were located within the resulting volumetric parcels. To improve on this, unassigned samples located less than 2 mm to a region boundary were additionally included to avoid exclusions due to registration misalignment, resulting in a final AHBA sample coverage of 91%. Gene expression values for each cortical parcel were estimated as the median gene expression of all the samples assigned to the parcel from all six donors. Expression values had very different scales across genes, so each gene expression value was normalized by taking a *Z*-score across regions. The normalised gene expression values were compiled as a (68 × 20,467) matrix that comprised transcriptional measurements for all 20,467 genes in the genome at each of 68 cortical regions.

Partial Least Squares analysis

Despite the high dimensionality on the transcriptomic data it is important to note that many of the genes have correlated expression patterns and this correlation structure severely reduces the effective degrees of freedom. To understand this, one may consider the extreme example where the dataset would consist of 20,000 identical (or perfectly correlated) genes – this would naturally be unlikely to reconstruct the 68-dimensional SRM map very well. In reality, the transcriptomic data are not so tightly (but still very highly) correlated. We note that this multicollinearity can be problematic for some statistical methods but the Partial Least Squares (PLS) method we use here was selected specifically for its ability to handle large datasets with high degrees on collinearity.

Supplementary Results

Protein-protein interaction network enrichment

We used the software STRING (version 10.5) to plot the protein-protein interaction network amongst the 213 genes of interest and to overlay gene set enrichments onto the resulting network (https://version-10-5.string-db.org/cgi/network.pl?taskId=RMpA04wbWG8k). Figure S11 shows enriched GO Cellular Component terms and highlights in colour individual genes/proteins from the PPI network which are implicated in these enriched GO terms. The significantly enriched terms (after FDR correction) include some general terms such as “cytoplasm” or “cell part” and neuron-specific terms such as “synapse”, “neuron part” and “myelin sheath”. A similar analysis for significantly enriched GO Biological Process terms includes again general terms such as “transport” and terms more specific to neuronal function such as “synaptic transmission”.

Replication using only left hemisphere data

The gene expression dataset provided by AHBA includes two subjects with data across both hemispheres and four subjects with data only measured in the left hemisphere. This sampling strategy was chosen due to the similarity of gene expression between hemispheres. By mirroring the samples across hemispheres, we artificially duplicated the gene expression data. When we restricted the schizophrenia-enrichment analysis only to the left hemisphere, we found that positively weighted genes on PLS were significantly enriched for down-regulated genes (Gandal, $P<10^{-4}$; Fromer, $P=0.004$) and negatively weighted genes on PLS were significantly enriched for up-regulated genes according to Gandal ($P<10^{-4}$) but only a trend was observed for Fromer ($P=0.059$).

Supplementary Figures

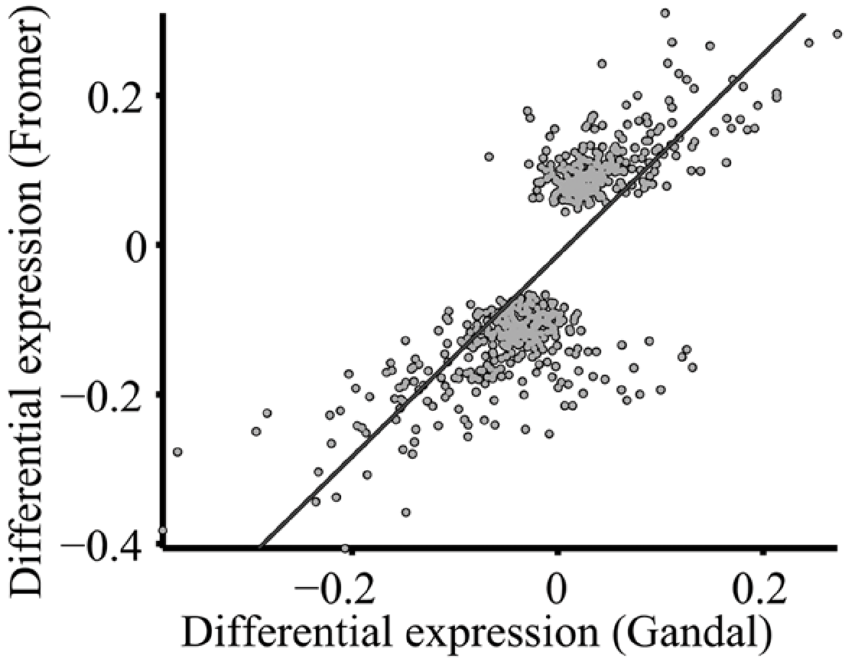

Figure S1. Correlation between differential *post-mortem* gene expression in schizophrenia defined by Gandal and Fromer (ρ = 0.76, $P < 10^{-129}$, df=641). Script used to generate this figure is available at:
https://github.com/RafaelRomeroGarcia/Schizotypy_MT_geneExp/tree/master/Code_for_Figure_S1

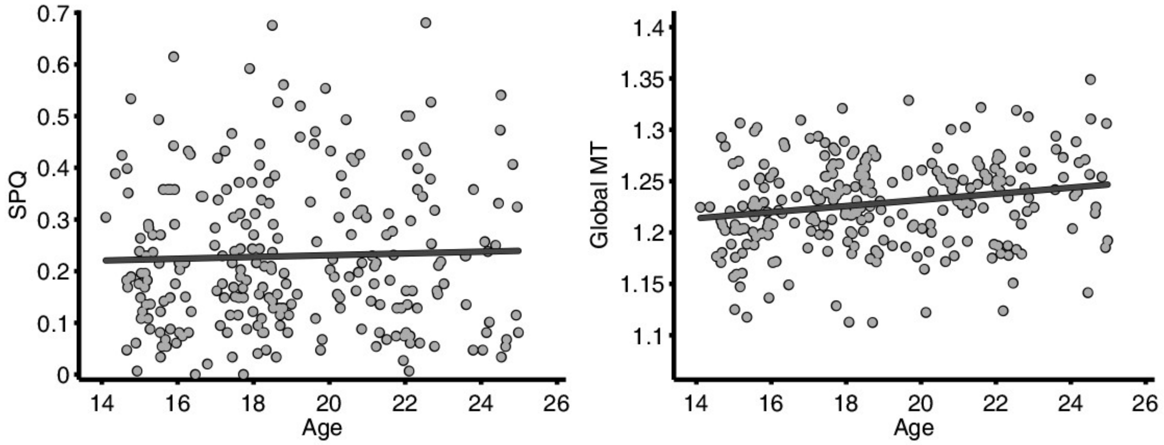

Figure S2. Scatterplots of the associations between age (x-axis) and total SPQ (left, $R^2 < 10^{-3}$, P=0.69, df = 247) and global MT (right, R^2 = 0.04, P=0.002, df = 247).

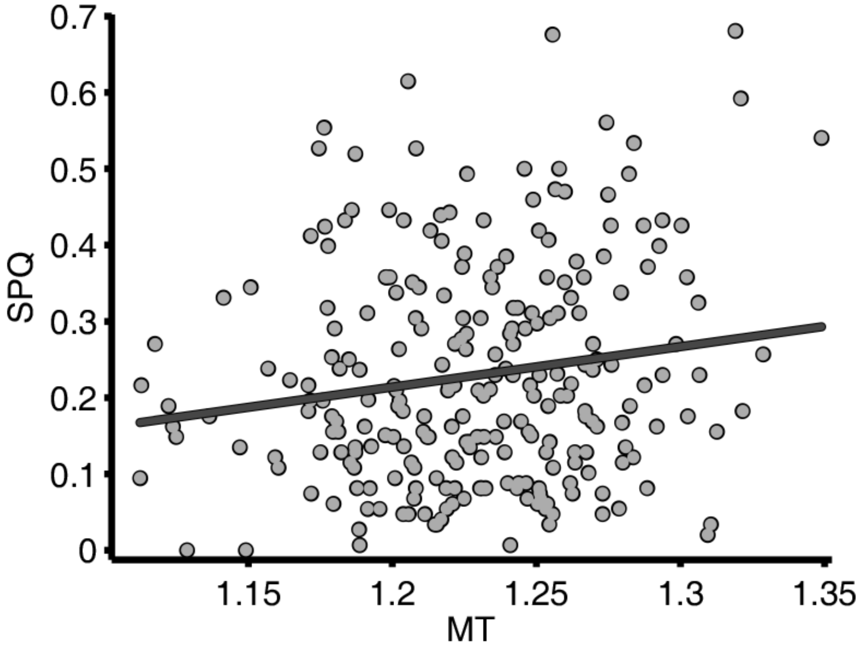

Figure S3. Scatterplot of total SPQ score (y-axis) versus global MT (averaged across regions) for 248 participants (R^2 = 0.02; P = 0.015; df = 247).

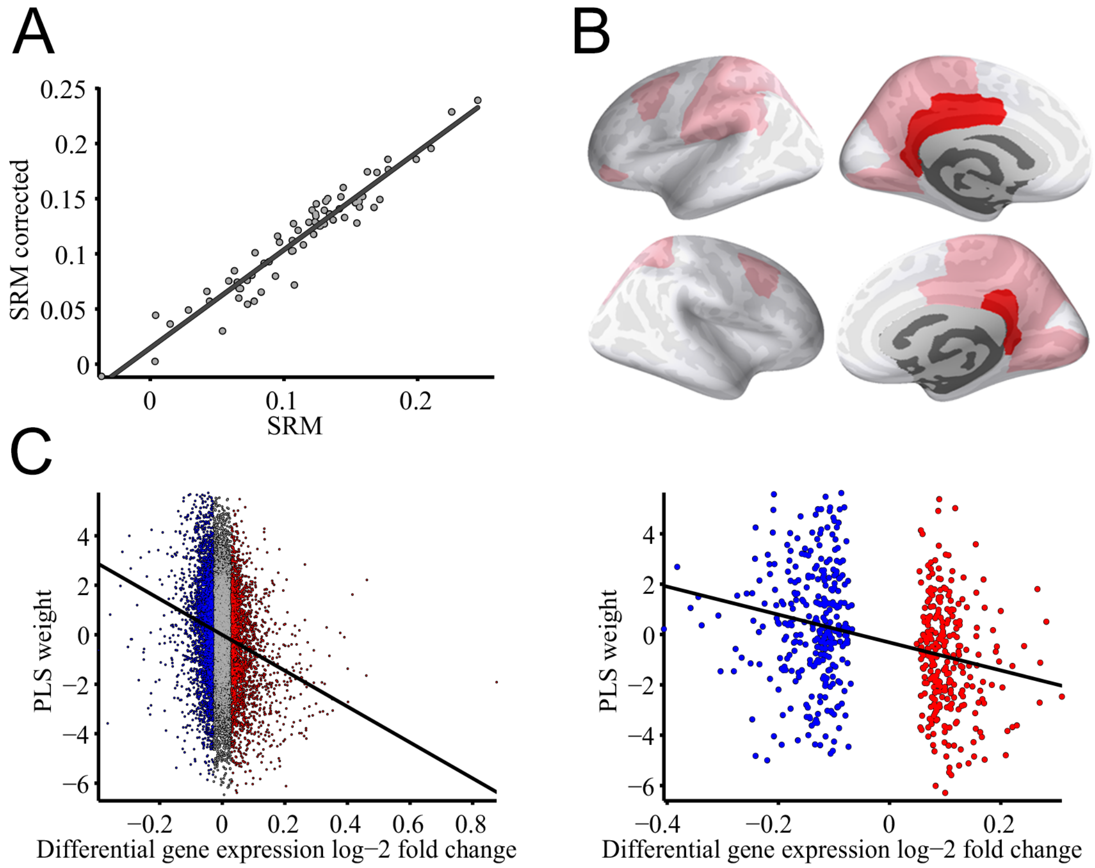

Figure S4. Schizotypy-related magnetization (SRM) after regressing out the effect of age, gender, scanning site, socio-economic status and total brain volume. (a) Scatterplot of SRM before and after removing the effect of the covariates (R^2 = 0.92, P < 10^{-37}, df = 66). (b) Cortical surface maps highlighting areas where total SPQ score was significantly positively correlated with regional MT after controlling for age, gender, scanning site, socio-economic status and total volume by regression: pink regions had nominally significant schizotypy-related magnetization (SRM) (two-tailed P < 0.05); red regions had significant SRM controlled for multiple comparisons over 68 cortical regions tested (FDR < 0.05). (c) The weight of each gene on the first PLS component calculated from SRM after regressing out age, gender and total brain volume was significantly negatively correlated with differential gene expression post mortem in schizophrenia according to prior data reported by Gandal et al (2018) (Spearman's rank correlation, ρ = -0.16, Bonferroni-corrected $P_{adj} < 10^{-61}$, df = 11111) and by Fromer et al (2016) (Spearman's rank correlation, ρ = -0.29, $P_{adj} < 10^{-12}$, df = 586; for this dataset only significantly different expression values have been reported (10)).

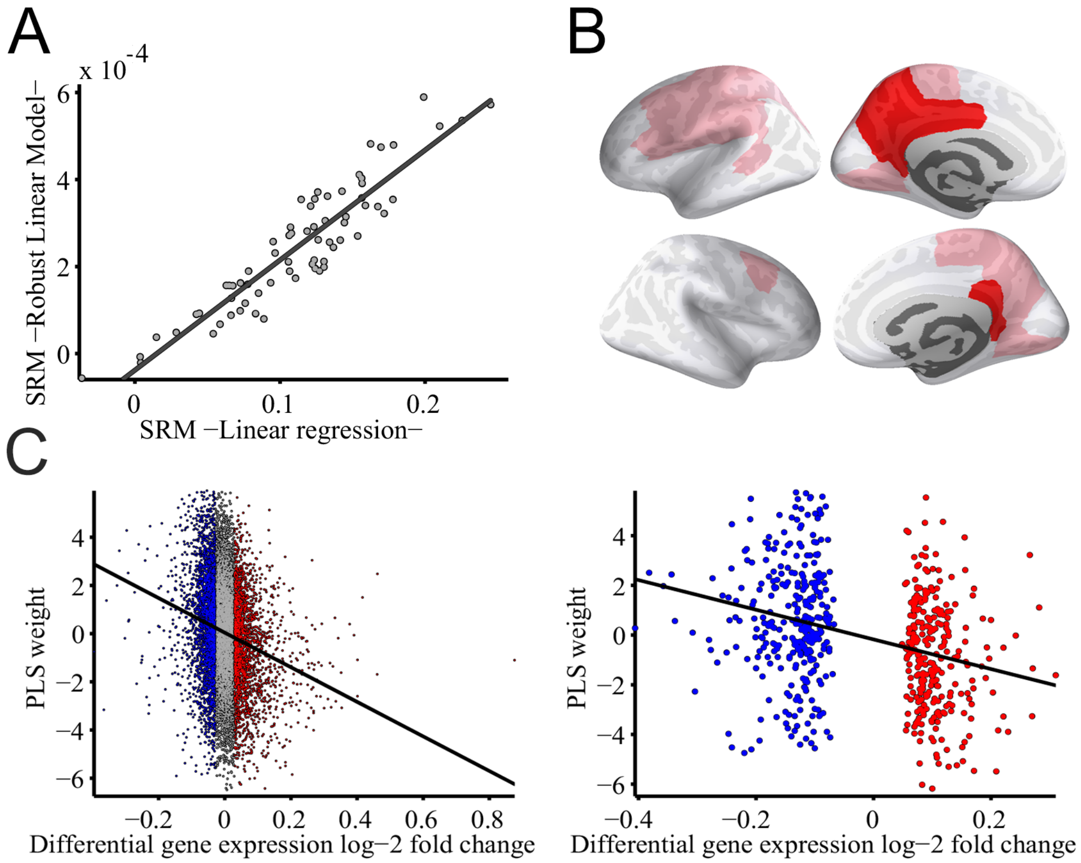

Figure S5. Schizotypy-related magnetization (SRM) calculated using robust linear models. (a) Scatterplot of SRM calculated using linear regression and robust linear models (R^2 = 0.85, P < 10^{-28}, df = 66). (b) Cortical surface maps highlighting areas where total SPQ score was significantly positively correlated with regional MT according to the robust linear regression: pink regions had nominally significant schizotypy-related magnetization (SRM) (two-tailed P < 0.05); red regions had significant SRM controlled for multiple comparisons over 68 cortical regions tested (FDR < 0.05). (c) The weight of each gene on the first PLS component calculated from SRM was significantly negatively correlated with differential gene expression post mortem in schizophrenia according to prior data reported by Gandal et al (2018) (Spearman's rank correlation, ρ = -0.18, Bonferroni-corrected $P_{adj} < 10^{-80}$, df = 11111) and by Fromer et al (2016) (Spearman's rank correlation, ρ = -0.28, $P_{adj} < 10^{-11}$, df = 586; for this dataset only significantly different expression values have been reported (10)).

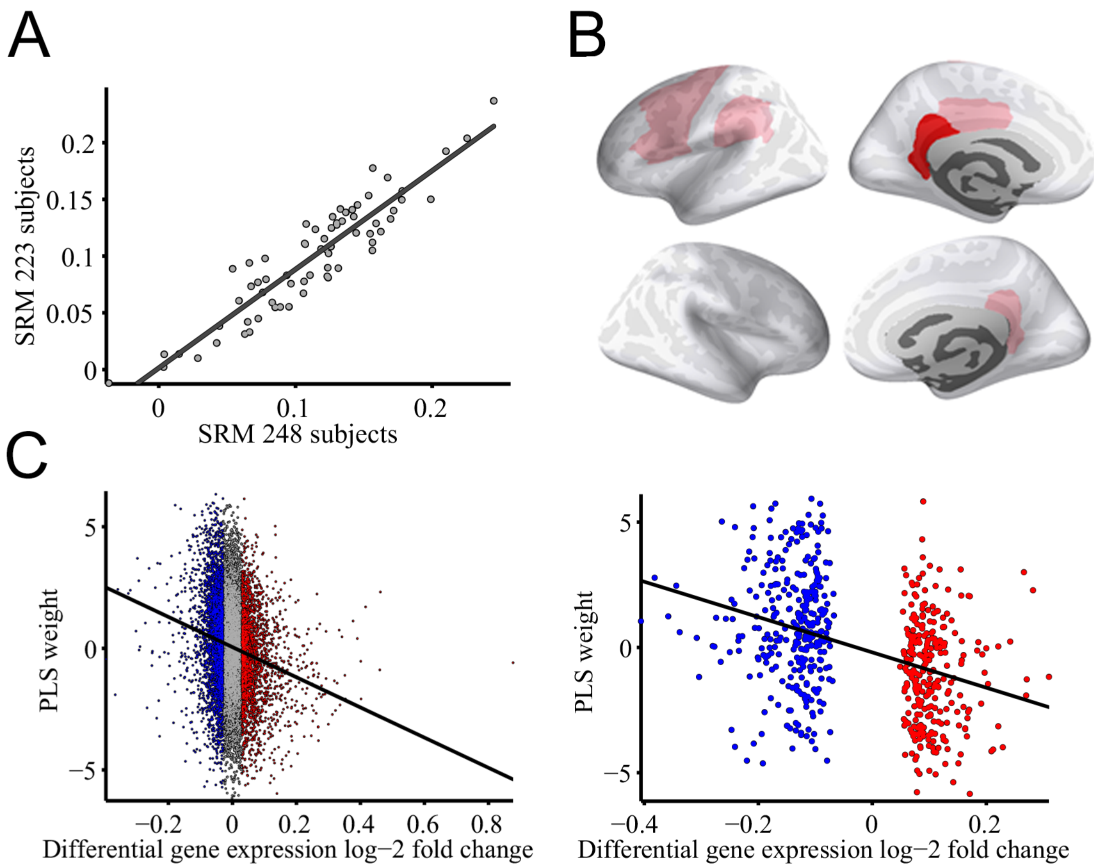

Figure S6. Schizotypy-related magnetization (SRM) calculated after removing the top 10% of the subject with worst image quality. (a) Scatterplot of SRM calculated with the original cohort 248 participant and SRM computed after removing those 25 images with highest Euler index (R^2 = 0.85, P < 10^{-28}, df = 66). (b) Cortical surface maps highlighting areas where total SPQ score calculated using 248 participants was significantly positively correlated with regional MT: pink regions had nominally significant schizotypy-related magnetization (SRM) (two-tailed P < 0.05); red regions had significant SRM controlled for multiple comparisons over 68 cortical regions tested (FDR < 0.05). (c) The weight of each gene on the first PLS component calculated from SRM was significantly negatively correlated with differential gene expression post mortem in schizophrenia according to prior data reported by Gandal et al (2018) (Spearman's rank correlation, ρ = -0.16, Bonferroni-corrected P_{adj} < 10^{-62}, df = 11111) and by Fromer et al (2016) (Spearman's rank correlation, ρ = -0.36, P_{adj} < 10^{-20}, df = 586; for this dataset only significantly different expression values have been reported (10)).

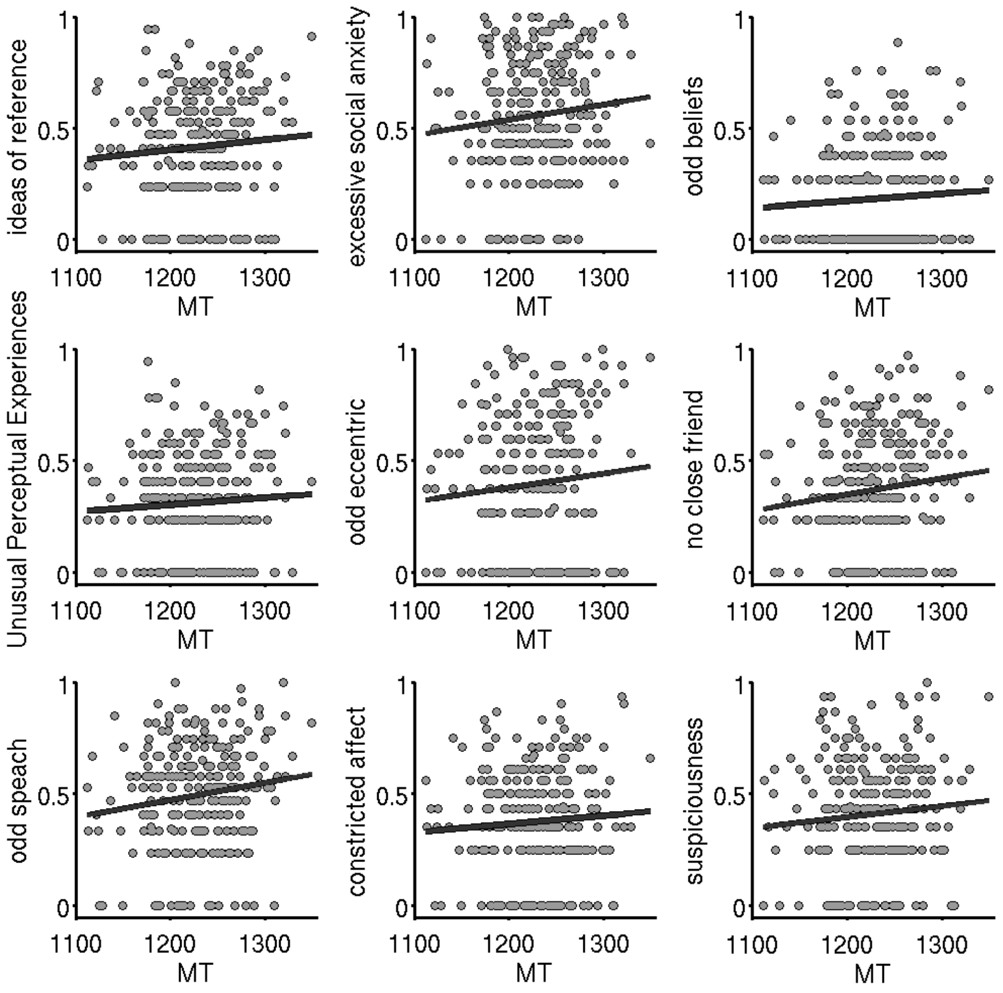

Figure S7. Association between global MT (averaged across all regions) and SPQ subscales for 248 participants. None of the correlations were significant after FDR correction (ideas of reference R^2=0.013, P=0.17; excessive social anxiety R^2=0.011, P=0.19; odd beliefs R^2=0.008, P=0.24; unusual perceptual experiences R^2=0.005, P=0.28; odd eccentric R^2=0.023, P=0.06; no close friend R^2=0.024, P=0.06; odd speech R^2=0.022, P=0.06; constricted affect R^2=0.005, P=0.27; suspiciousness R^2=0.005, P=0.27; df = 247).

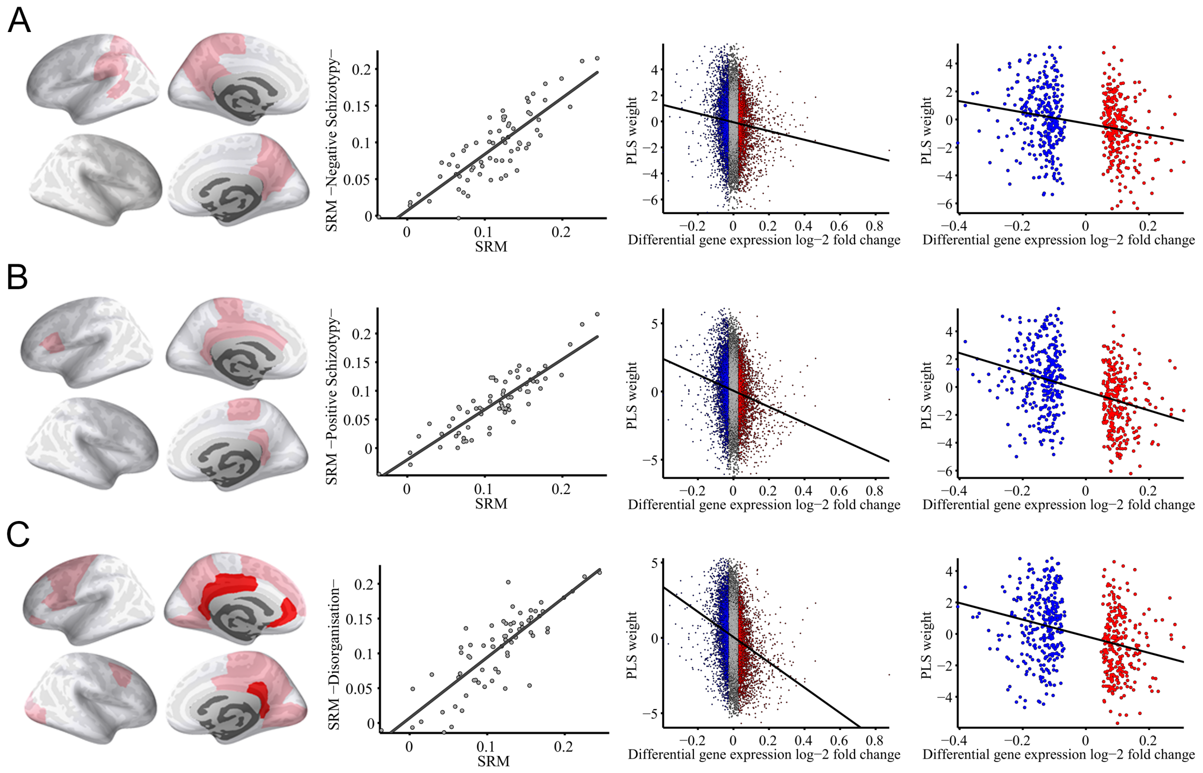

Figure S8. Association between MT and the three factor models of schizotypy: (a) Negative Schizotypy factor, (b) Positive Schizotypy factor and (c) Disorganisation. Cortical surface maps highlighting areas where each factor score was significantly positively correlated with regional MT (pink, two-tailed $P < 0.05$; red, FDR < 0.05; First column). Scatterplot of SRM calculated from total SPQ compared with SRM based on each individual factor ($R^2 = 0.70$, $P < 10^{-18}$ for Negative Schizotypy, $R^2 = 0.77$, $P < 10^{-22}$ for Positive Schizotypy, $R^2 = 0.69$, $P < 10^{-17}$ for Disorganisation; df=67; Second column). The weight of each gene on the first PLS calculated from SRM based on each individual factor was correlated with differential gene expression post mortem in schizophrenia (Negative Schizotypy: $\rho = -0.06$, $P_{adj} < 10^{-10}$ and $\rho = -0.24$, $P_{adj} < 10^{-8}$ for Gandal -third column- and Fromer -fourth column- differential expression respectively; Positive Schizotypy: $\rho = -0.13$, $P_{adj} < 10^{-47}$ and $\rho = -0.36$, $P_{adj} < 10^{-18}$; Disorganisation: $\rho = -0.22$, $P_{adj} < 10^{-121}$ and $\rho = -0.28$, $P_{adj} < 10^{-12}$).

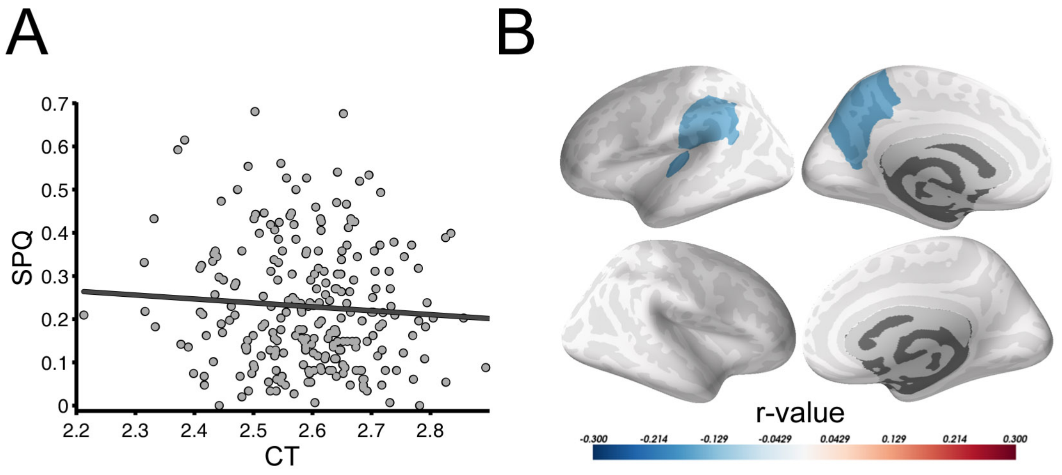

Figure S9. Schizotypy-dependent cortical thickness of parietal lobe. (a) Scatterplot of SPQ total score versus global mean CT (averaged over all regions; $R^2 = 0.005$; $P = 0.27$; df = 247). (b) Cortical surface maps highlighting cortical areas where total SPQ score was significantly negatively correlated with regional CT: blue regions had nominally significant SPQ/CT correlation ($P<0.025$, uncorrected); however, none of these regional associations survived correction for multiple comparisons.

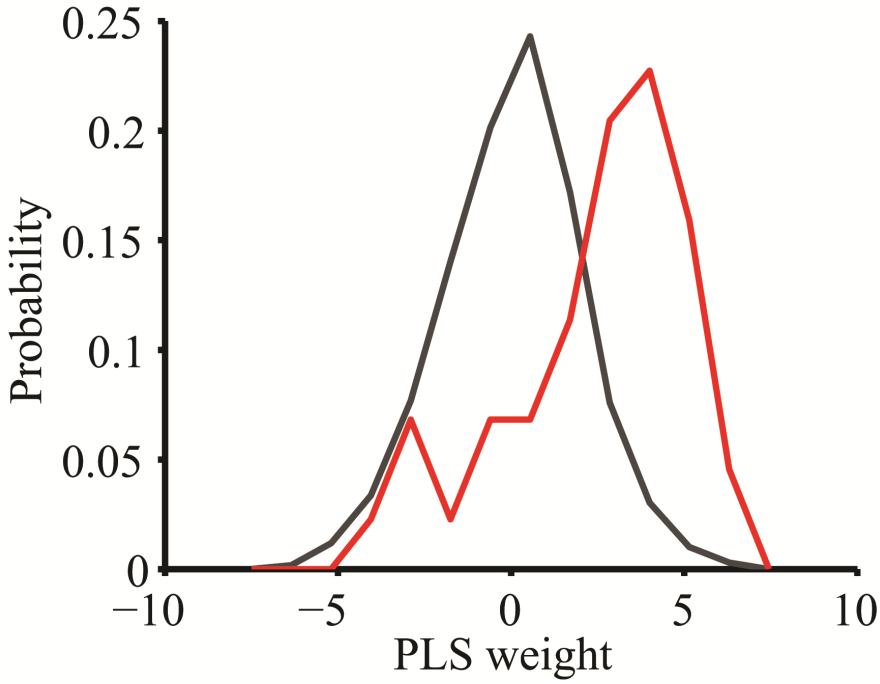

Figure S10. Distribution of PLS weights calculated from SRM for all genes (black) and genes that correlate with macroscale disconnectivity in schizophrenia as reported by (11). The 156 genes showing the strongest correlation with white-matter connectivity changes in schizophrenia described by (11) had significantly higher PLS weight that the rest of genes (t-test, $P < 10^{-14}$) and were more frequently ranked at the top of the PLS component than expected by chance (permutation test, $P < 10^{-4}$).

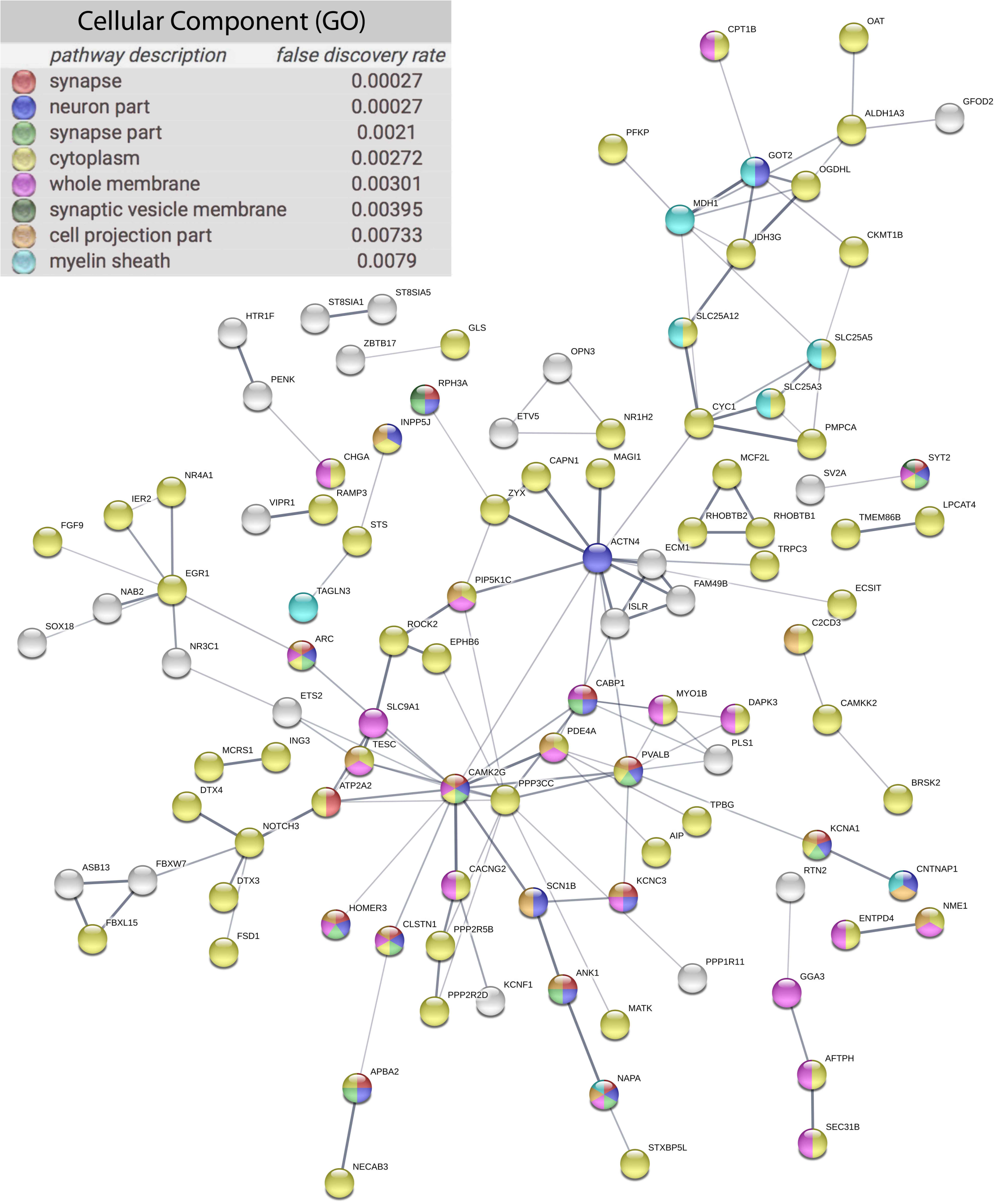

Figure S11. Protein-protein interaction network for the set of 213 proteins coded by genes associated with both schizotypy-related magnetization and post-mortem brain transcription in schizophrenia. Nodes represent genes that were both (i) down-regulated in brain tissue from 159 patients with schizophrenia (12); and (ii) positively weighted on the PLS component most strongly associated with schizotypy-related myelination in 248 healthy adolescents. Edges represent known protein-protein interactions and weights are proportional to the STRING confidence score > 0.4 (13). Proteins are coloured by their participation in the most highly enriched GO cellular component terms; all *P*-values are reported after FDR correction. See https://version-10-5.string-db.org/cgi/network.pl?taskId=RMpA04wbWG8k for a full interactive map of PPI network.

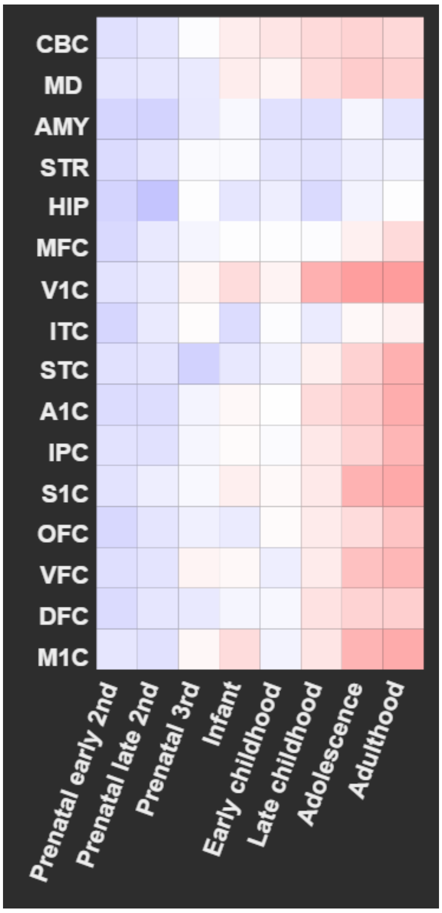

Figure S12. Expression changes of positively-weighted SRM genes across developmental stages. Average gene expression values of positively-weighted SRM calculated form the Developing Human Transcriptome included in the BrainSpan Atlas (http://brainspan.org./static/home). CBC, cerebellar cortex; MD, mediodorsal nucleus of the thalamus; AMY, amygdala; STR, striatum; HIP, hippocampus; MFC, medial prefrontal cortex; V1C, primary visual (V1) cortex; ITC, inferior temporal cortex; STC, superior temporal cortex; A1C, primary auditory (A1) cortex; IPC, posterior inferior parietal cortex; S1C, primary somatosensory (S1) cortex; OFC, orbital prefrontal cortex; VFC, ventrolateral prefrontal cortex; DFC, dorsolateral prefrontal cortex; M1C, primary motor (M1) cortex. Figure generated using BrainScope (https://brainscope.lumc.nl/brainscope).

Supplementary Tables

Age (yrs)	Gender	N	IR	ESA	OBMT	UPE	OEB	NCF	OS	CA	S	Total SPQ
14 to 15	M	27	0.14	0.21	0.02	0.10	0.18	0.07	0.18	0.07	0.18	**0.09**
	F	25	0.22	0.32	0.02	0.05	0.17	0.07	0.19	0.05	0.12	**0.08**
16 to 17	M	25	0.14	0.23	0.06	0.09	0.26	0.12	0.20	0.11	0.14	**0.10**
	F	26	0.17	0.29	0.08	0.07	0.13	0.07	0.16	0.06	0.13	**0.09**
18 to 19	M	25	0.18	0.26	0.03	0.10	0.16	0.11	0.20	0.14	0.23	**0.12**
	F	27	0.15	0.30	0.04	0.06	0.12	0.11	0.13	0.06	0.15	**0.08**
20 to 21	M	21	0.10	0.27	0.05	0.12	0.33	0.06	0.21	0.10	0.08	**0.10**
	F	22	0.11	0.20	0.04	0.07	0.13	0.04	0.14	0.04	0.16	**0.07**
22 to 25	M	27	0.10	0.20	0.03	0.06	0.19	0.17	0.20	0.12	0.12	**0.09**
	F	23	0.21	0.40	0.06	0.06	0.25	0.13	0.21	0.12	0.17	**0.12**

Table S1: Schizotypal Personality Questionnaire: total score and subscale scores stratified by age and sex. IR = Ideas of References; ESA = Excessive Social Anxiety; OBMT = Odd Beliefs or Magical Thinking; UPE = Unusual Perceptual Experiences; OEB = Odd or Eccentric Behavior; NCF = No Close Friends; OS = Odd Speech; CA = Constricted Affect; S = Suspiciousness.

	Number of genes
Astrocyte	280
Endothelial	177
GabaPV	15
GabaRelnCalb	15
GabaVIPReln	33
Layer 2 3 Pyra	4
Layer 4 Pyra	5
Layer 6a Pyra	6
Layer 6b Pyra	9
Microglia	284
Microglia_activation	118
Microglia_deactivation	199
Oligo	152
OligoPrecursors	185
Pyramidal	35
PyramidalCorticoThalam	2
Pyramidal_Glt_25d2	3
Pyramidal_S100a10	2

Table S2. Cell-type expression profiles defined by (10). Each cell-type was associated with the differential expression of a subset of genes.

	Gandal Up-reg	Gandal Down-reg	Fromer Up-reg	Fromer Down-reg
**Gandal Up-reg**	845	0	39	11
**Gandal Down-reg**		1177	4	76
**Fromer Up-reg**			304	0
**Fromer Down-reg**				345

Table S3. Number of schizophrenia-related genes overlapping between lists of up and down-regulated genes defined by (12) and up and down-regulated genes defined by (14).

Region	*r*-value	*p*-value
LH isthmuscingulate	0.24506	9.65E-05
RH isthmuscingulate	0.22557	0.000343
LH posteriorcingulate	0.21008	0.000872
LH precuneus	0.19896	0.001639
LH supramarginal	0.17812	0.004901
LH paracentral	0.1778	0.004981
RH caudalmiddlefrontal	0.17176	0.0067
RH lingual	0.1696	0.007432
LH precentral	0.16741	0.00825
RH precuneus	0.16242	0.01041
LH postcentral	0.15889	0.01223
LH parsopercularis	0.15656	0.013577
LH superiorparietal	0.15644	0.01365
LH lingual	0.15627	0.013755
LH bankssts	0.15461	0.014801
LH caudalmiddlefrontal	0.15349	0.015552
RH precentral	0.14538	0.022019
RH paracentral	0.14435	0.022985
RH posteriorcingulate	0.14266	0.024656
LH caudalanteriorcingulate	0.14153	0.025827
RH medialorbitofrontal	0.13686	0.031203
LH superiorfrontal	0.13413	0.03476
RH pericalcarine	0.13276	0.036671
RH superiorparietal	0.1312	0.038951
LH lateralorbitofrontal	0.13029	0.04034
RH lateralorbitofrontal	0.13029	0.040341
LH rostralanteriorcingulate	0.12723	0.045321
RH parstriangularis	0.12629	0.046953
LH inferiorparietal	0.12592	0.047608
LH parsorbitalis	0.12421	0.050721
RH supramarginal	0.12389	0.05134
LH rostralmiddlefrontal	0.12364	0.051801
LH transversetemporal	0.12332	0.052415
RH superiorfrontal	0.12224	0.054534
RH lateraloccipital	0.12104	0.056973
RH postcentral	0.11864	0.0621
RH insula	0.11452	0.071826
RH parsorbitalis	0.1107	0.081872
LH insula	0.10785	0.090124
LH pericalcarine	0.10716	0.092208
LH parstriangularis	0.1067	0.093625
LH middletemporal	0.10632	0.094807
LH cuneus	0.1058	0.096429

RH fusiform	0.096602	0.12923
RH inferiorparietal	0.095275	0.1346
RH rostralmiddlefrontal	0.093595	0.14164
LH frontalpole	0.088892	0.16285
LH parahippocampal	0.085186	0.18117
RH entorhinal	0.083059	0.19235
LH lateraloccipital	0.078353	0.21886
RH parsopercularis	0.077761	0.22237
LH medialorbitofrontal	0.076083	0.23254
LH superiortemporal	0.072809	0.25332
RH rostralanteriorcingulate	0.072621	0.25455
RH bankssts	0.067464	0.28994
RH cuneus	0.066513	0.29681
RH caudalanteriorcingulate	0.066168	0.29932
LH inferiortemporal	0.065014	0.30784
RH inferiortemporal	0.062987	0.32321
RH temporalpole	0.058726	0.35708
RH frontalpole	0.054053	0.39669
RH transversetemporal	0.044336	0.48704
LH fusiform	0.042373	0.50656
LH entorhinal	-0.03653	0.56697
RH parahippocampal	0.028528	0.65481
RH middletemporal	0.014899	0.8154
LH temporalpole	0.004035	0.94959
RH superiortemporal	0.003549	0.95566

Table S4. Correlation between total SPQ and regional MT. Red rows highlight significant correlations (*P*<0.05, FDR corrected).

Gene Name	Schizophrenia-related list	PLS1 *Z*-score	PLS1 Adjusted *P*-value	PLS1 Ranking out of 20,647	Inter-regional correlation	Inter-regional *P*-value
IER2	GDR	6.41	1.00E-06	2	0.43	2.26E-02
ESRRA	GDR	6.12	1.00E-06	9	0.50	7.91E-03
CAMK2G	GDR	6.03	1.00E-06	22	0.47	1.18E-02
PDE4A	GDR	6.03	1.00E-06	23	0.44	1.94E-02
TRPC3	GDR	5.99	1.00E-06	25	0.36	6.82E-02
ESRRG	GDR	5.99	1.00E-06	26	0.43	2.23E-02
ANK1	GDR	5.96	1.00E-06	27	0.45	1.76E-02
ECM1	GDR	5.94	1.00E-06	28	0.45	1.82E-02
SCN1B	GDR	5.92	1.00E-06	29	0.40	3.81E-02
KCNA1	GDR	5.76	2.00E-06	38	0.45	1.74E-02
SLC25A5	GDR	5.74	2.00E-06	41	0.33	9.82E-02
ING3	GDR	5.71	3.00E-06	45	0.37	6.02E-02
KCNC3	GDR	5.68	3.00E-06	48	0.42	2.92E-02
SEMA7A	GDR	5.63	4.00E-06	54	0.38	4.90E-02
FAM131B	GDR	5.51	5.00E-06	75	0.49	9.25E-03
SLC38A1	GDR	5.45	6.00E-06	82	0.45	1.74E-02
RHOBTB2	GDR	5.39	8.00E-06	90	0.45	1.88E-02
GLS2	GDR	5.38	8.00E-06	91	0.38	5.34E-02
RFX5	GDR	5.37	9.00E-06	94	0.43	2.60E-02
HTR1F	GDR	5.33	1.00E-05	99	0.43	2.48E-02
SPHK2	GDR	5.32	1.00E-05	101	0.48	9.25E-03
ETS2	GDR	5.28	1.30E-05	106	0.34	8.44E-02
SYT2	GDR	5.16	2.10E-05	116	0.38	4.80E-02
CRABP1	GDR	5.13	2.40E-05	121	0.34	9.52E-02
DNAJC4	GDR	5.12	2.60E-05	122	0.36	6.68E-02
CNTNAP1	GDR	5.11	2.70E-05	126	0.43	2.62E-02
SCRT1	GDR	5.07	3.10E-05	132	0.42	2.89E-02
OGDHL	GDR	5.00	4.00E-05	143	0.36	7.19E-02
FGF9	GDR	4.99	4.20E-05	150	0.40	3.97E-02
TRAF3	GDR	4.97	4.30E-05	155	0.48	9.95E-03
DCUN1D2	GDR	4.94	5.00E-05	163	0.40	3.97E-02
ST8SIA5	GDR	4.90	5.80E-05	167	0.37	6.02E-02
HECA	GDR	4.90	6.00E-05	169	0.40	3.63E-02
GFRA2	GDR	4.86	7.00E-05	173	0.43	2.23E-02
SLC7A8	GDR	4.85	7.30E-05	178	0.48	9.68E-03
CKMT1B	GDR	4.84	7.40E-05	180	0.37	5.92E-02
OSBPL3	GDR	4.82	8.00E-05	183	0.45	1.82E-02
SYT12	GDR	4.76	1.03E-04	199	0.41	3.15E-02
CABP1	GDR	4.74	1.08E-04	207	0.39	4.40E-02
LUZP1	GDR	4.74	1.08E-04	208	0.36	7.23E-02
PLXDC1	GDR	4.73	1.09E-04	210	0.41	3.19E-02
RAB11FIP5	GDR	4.66	1.41E-04	237	0.34	9.60E-02

ASB13	GDR	4.65	1.44E-04	239	0.36	6.51E-02
SLC25A12	GDR	4.63	1.55E-04	240	0.33	9.82E-02
DDHD2	GDR	4.63	1.59E-04	241	0.42	2.66E-02
ZMAT4	GDR	4.61	1.68E-04	248	0.37	6.27E-02
MAGI1	GDR	4.59	1.77E-04	253	0.28	1.82E-01
DPP8	GDR	4.58	1.84E-04	256	0.35	8.41E-02
PRDM1	GDR	4.58	1.86E-04	260	0.45	1.81E-02
SV2A	GDR	4.58	1.87E-04	261	0.37	5.68E-02
SLC20A1	GDR	4.55	2.07E-04	269	0.46	1.44E-02
ARC	GDR	4.55	2.09E-04	270	0.40	3.92E-02
OSBP2	GDR	4.54	2.10E-04	271	0.37	6.24E-02
FBXW7	GDR	4.49	2.55E-04	282	0.38	5.36E-02
CPT1B	GDR	4.49	2.64E-04	285	0.37	5.68E-02
CHRD	GDR	4.45	3.03E-04	295	0.32	1.12E-01
SV2C	GDR	4.43	3.26E-04	299	0.34	8.44E-02
NR3C1	GDR	4.40	3.58E-04	315	0.40	3.55E-02
CAMKK2	GDR	4.37	3.90E-04	323	0.39	4.57E-02
DEPDC5	GDR	4.37	3.91E-04	324	0.37	5.71E-02
ZYX	GDR	4.35	4.23E-04	335	0.38	4.97E-02
ATG16L1	GDR	4.35	4.24E-04	336	0.29	1.73E-01
HHAT	GDR	4.33	4.51E-04	344	0.55	4.73E-03
ROCK2	GDR	4.33	4.54E-04	345	0.34	8.75E-02
PNMA3	GDR	4.31	4.70E-04	356	0.33	1.03E-01
ST8SIA1	GDR	4.31	4.70E-04	358	0.31	1.25E-01
POU6F1	GDR	4.30	4.85E-04	365	0.31	1.28E-01
BRSK2	GDR	4.28	5.16E-04	374	0.34	8.43E-02
LPCAT4	GDR	4.27	5.30E-04	380	0.32	1.23E-01
HR	GDR	4.27	5.34E-04	382	0.37	5.46E-02
NARS	GDR	4.24	5.80E-04	398	0.33	1.05E-01
ATRNL1	GDR	4.22	6.26E-04	406	0.33	1.04E-01
NUAK1	GDR	4.22	6.26E-04	407	0.33	9.92E-02
ALDH1A3	GDR	4.22	6.28E-04	410	0.29	1.73E-01
PPP3CC	GDR	4.15	7.93E-04	440	0.25	2.67E-01
ISLR	GDR	4.13	8.28E-04	449	0.38	5.08E-02
TMEM127	GDR	4.07	9.98E-04	482	0.27	2.24E-01
C6orf106	GDR	4.02	1.19E-03	501	0.33	9.73E-02
PFKP	GDR	4.01	1.22E-03	508	0.32	1.14E-01
FNDC4	GDR	4.01	1.23E-03	509	0.30	1.53E-01
PVALB	GDR	4.01	1.25E-03	511	0.31	1.36E-01
RUNDC3B	GDR	3.99	1.30E-03	520	0.25	2.61E-01
ZNF385D	GDR	3.98	1.36E-03	529	0.36	6.93E-02
RHOBTB1	GDR	3.97	1.40E-03	533	0.36	7.10E-02
EPHB6	GDR	3.93	1.59E-03	548	0.42	2.92E-02
LGI2	GDR	3.93	1.60E-03	556	0.33	1.07E-01
LAPTM4B	GDR	3.92	1.65E-03	559	0.30	1.49E-01
GOT2	GDR	3.88	1.85E-03	579	0.27	2.02E-01

ANKS1A	GDR	3.85	2.05E-03	597	0.27	2.14E-01
SCAMP5	GDR	3.84	2.12E-03	605	0.32	1.18E-01
IDH3G	GDR	3.83	2.16E-03	608	0.31	1.26E-01
STAU2	GDR	3.82	2.22E-03	624	0.37	6.13E-02
ANXA6	GDR	3.82	2.22E-03	626	0.25	2.62E-01
SMYD2	GDR	3.79	2.44E-03	646	0.33	1.04E-01
PITPNA	GDR	3.77	2.58E-03	659	0.29	1.70E-01
STS	GDR	3.74	2.76E-03	679	0.23	3.02E-01
RAMP3	GDR	3.74	2.76E-03	681	0.42	3.10E-02
PCGF1	GDR	3.73	2.83E-03	688	0.33	1.03E-01
VIPR1	GDR	3.69	3.30E-03	716	0.30	1.58E-01
RTN2	GDR	3.68	3.35E-03	722	0.39	4.38E-02
TPBG	GDR	3.66	3.50E-03	732	0.33	1.02E-01
MED22	GDR	3.66	3.50E-03	733	0.24	2.85E-01
MAP4K2	GDR	3.66	3.51E-03	736	0.33	1.00E-01
PKNOX2	GDR	3.66	3.57E-03	741	0.28	1.84E-01
PHLDA2	GDR	3.65	3.60E-03	742	0.30	1.58E-01
HOMER3	GDR	3.65	3.62E-03	744	0.25	2.59E-01
GFOD2	GDR	3.64	3.77E-03	747	0.32	1.10E-01
DTX3	GDR	3.63	3.90E-03	754	0.25	2.67E-01
OAT	GDR	3.63	3.92E-03	755	0.32	1.17E-01
NR4A1	GDR	3.61	4.13E-03	770	0.30	1.54E-01
NRN1	GDR	3.60	4.24E-03	779	0.27	2.21E-01
HS3ST1	GDR	3.59	4.40E-03	788	0.22	3.35E-01
OPN3	GDR	3.59	4.40E-03	789	0.39	4.54E-02
ICA1	GDR	3.56	4.78E-03	809	0.28	1.97E-01
CHGA	GDR	3.55	4.81E-03	812	0.33	9.91E-02
GLS	GDR	3.49	5.75E-03	857	0.24	2.89E-01
SRA1	GDR	3.48	5.93E-03	863	0.25	2.62E-01
CLSTN1	GDR	3.48	5.99E-03	866	0.29	1.67E-01
ETV5	GDR	3.45	6.58E-03	883	0.33	1.08E-01
BID	GDR	3.43	7.02E-03	899	0.17	5.08E-01
NAB2	GDR	3.42	7.20E-03	912	0.28	1.80E-01
EXTL2	GDR	3.40	7.60E-03	922	0.32	1.20E-01
C16orf45	GDR	3.39	7.69E-03	930	0.25	2.55E-01
KCNK12	GDR	3.35	8.55E-03	975	0.25	2.63E-01
PLS1	GDR	3.33	9.04E-03	986	0.32	1.14E-01
CRTAC1	GDR	3.33	9.06E-03	987	0.32	1.12E-01
TAPBP	GDR	3.33	9.21E-03	990	0.29	1.72E-01
SERGEF	GDR	3.32	9.28E-03	994	0.39	4.67E-02
SLC9A1	GDR	3.32	9.45E-03	1001	0.27	2.11E-01
JOSD1	GDR	3.31	9.49E-03	1005	0.30	1.49E-01
NECAB3	GDR	3.31	9.63E-03	1016	0.21	3.58E-01
ABCC5	GDR	3.30	9.82E-03	1025	0.26	2.49E-01
PEPD	GDR	3.30	9.83E-03	1030	0.21	3.73E-01
REEP2	GDR	3.29	9.95E-03	1038	0.26	2.33E-01

TMEM86B	GDR	3.27	1.06E-02	1049	0.29	1.77E-01
SLC25A3	GDR	3.26	1.09E-02	1064	0.26	2.35E-01
ZBTB17	GDR	3.25	1.11E-02	1068	0.32	1.08E-01
TESC	GDR	3.24	1.13E-02	1076	0.27	2.09E-01
C2CD3	GDR	3.24	1.13E-02	1079	0.28	1.98E-01
MCF2L	GDR	3.24	1.13E-02	1080	0.22	3.39E-01
PIP5K1C	GDR	3.24	1.15E-02	1082	0.31	1.34E-01
MAP3K13	GDR	3.23	1.17E-02	1089	0.29	1.66E-01
C8orf4	GDR	3.23	1.18E-02	1093	0.37	6.02E-02
ACTN4	GDR	3.22	1.21E-02	1103	0.27	2.18E-01
EGR1	GDR	3.22	1.21E-02	1106	0.27	2.03E-01
PPP2R5B	GDR	3.21	1.22E-02	1115	0.29	1.73E-01
INPP5J	GDR	3.21	1.23E-02	1119	0.25	2.72E-01
LRRC59	GDR	3.21	1.23E-02	1124	0.23	3.16E-01
RUSC2	GDR	3.20	1.24E-02	1125	0.30	1.42E-01
UBFD1	GDR	3.19	1.29E-02	1143	0.25	2.63E-01
LBH	GDR	3.18	1.31E-02	1153	0.33	1.02E-01
DUS1L	GDR	3.17	1.34E-02	1160	0.27	2.23E-01
NXPH3	GDR	3.17	1.35E-02	1165	0.28	1.79E-01
NOTCH3	GDR	3.15	1.41E-02	1188	0.32	1.09E-01
RWDD1	GDR	3.15	1.42E-02	1191	0.20	4.03E-01
GABRA4	GDR	3.15	1.42E-02	1193	0.29	1.66E-01
ATP2A2	GDR	3.14	1.45E-02	1199	0.23	3.19E-01
CACNG2	GDR	3.12	1.54E-02	1221	0.31	1.32E-01
SNCB	GDR	3.11	1.56E-02	1229	0.23	3.06E-01
STXBP5L	GDR	3.10	1.61E-02	1245	0.25	2.66E-01
TSPAN9	GDR	3.09	1.66E-02	1259	0.26	2.34E-01
ITIH5	GDR	3.06	1.76E-02	1285	0.25	2.60E-01
PPP2R2D	GDR	3.06	1.77E-02	1288	0.22	3.53E-01
C12orf49	GDR	3.05	1.83E-02	1298	0.28	1.94E-01
NDRG4	GDR	3.05	1.83E-02	1300	0.23	3.04E-01
SNRNP25	GDR	3.04	1.84E-02	1309	0.24	2.83E-01
ECSIT	GDR	3.03	1.90E-02	1330	0.28	1.89E-01
SHROOM2	GDR	3.01	2.01E-02	1352	0.23	3.15E-01
URM1	GDR	2.99	2.12E-02	1372	0.31	1.33E-01
DTX4	GDR	2.98	2.13E-02	1376	0.25	2.67E-01
MATK	GDR	2.98	2.16E-02	1383	0.28	1.94E-01
TRMU	GDR	2.94	2.35E-02	1427	0.29	1.77E-01
CAPN1	GDR	2.92	2.46E-02	1452	0.24	2.77E-01
WDR45	GDR	2.90	2.62E-02	1478	0.22	3.44E-01
RUSC1	GDR	2.87	2.77E-02	1506	0.25	2.70E-01
SEC31B	GDR	2.86	2.87E-02	1524	0.22	3.36E-01
MFGE8	GDR	2.86	2.89E-02	1530	0.22	3.55E-01
APBA2	GDR	2.85	2.91E-02	1539	0.21	3.84E-01
GGA3	GDR	2.84	3.01E-02	1567	0.20	4.14E-01
CCDC25	GDR	2.83	3.07E-02	1579	0.21	3.84E-01

CHST8		2.82	3.14E-02	1591	0.29	1.65E-01
MYO1B	GDR	2.81	3.18E-02	1596	0.25	2.73E-01
TNFAIP1	GDR	2.80	3.24E-02	1604	0.27	2.18E-01
ITM2A	GDR	2.80	3.25E-02	1605	0.18	4.66E-01
RPH3A	GDR	2.80	3.32E-02	1611	0.26	2.45E-01
CYC1	GDR	2.80	3.32E-02	1612	0.23	3.04E-01
SLC24A2	GDR	2.79	3.33E-02	1614	0.22	3.37E-01
PENK	GDR	2.79	3.34E-02	1617	0.21	3.73E-01
DAPK3	GDR	2.79	3.38E-02	1626	0.23	3.00E-01
PMPCA	GDR	2.78	3.43E-02	1631	0.26	2.34E-01
FAM49B	GDR	2.77	3.57E-02	1644	0.24	2.94E-01
NRSN2	GDR	2.76	3.62E-02	1650	0.31	1.32E-01
MTDH	GDR	2.75	3.68E-02	1665	0.24	2.94E-01
SPRY2	GDR	2.74	3.77E-02	1684	0.20	3.95E-01
FBXL15	GDR	2.73	3.81E-02	1691	0.24	2.98E-01
SOX18	GDR	2.73	3.85E-02	1697	0.25	2.53E-01
NAPA	GDR	2.72	3.91E-02	1703	0.23	3.11E-01
MDH1	GDR	2.72	3.93E-02	1704	0.20	3.93E-01
NME1	GDR	2.72	3.97E-02	1712	0.19	4.43E-01
ENTPD4	GDR	2.71	3.99E-02	1716	0.20	4.14E-01
KATNB1	GDR	2.71	4.01E-02	1723	0.21	3.67E-01
GDPD5	GDR	2.70	4.16E-02	1738	0.27	2.04E-01
KCNF1	GDR	2.69	4.21E-02	1741	0.29	1.78E-01
PPP1R11	GDR	2.69	4.22E-02	1743	0.24	2.79E-01
HS3ST2	GDR	2.69	4.26E-02	1747	0.28	1.85E-01
EDN3	GDR	2.68	4.35E-02	1763	0.18	4.54E-01
POSTN	GDR	2.67	4.37E-02	1768	0.18	4.56E-01
KCNJ8	GDR	2.67	4.37E-02	1772	0.27	2.23E-01
MPPED1	GDR	2.67	4.38E-02	1776	0.25	2.57E-01
C12orf10	GDR	2.66	4.47E-02	1792	0.22	3.35E-01
RPP25	GDR	2.66	4.47E-02	1793	0.19	4.22E-01
MRPS15	GDR	2.66	4.52E-02	1805	0.22	3.47E-01
MGST3	GDR	2.65	4.58E-02	1815	0.20	4.17E-01
FSD1	GDR	2.65	4.61E-02	1825	0.25	2.53E-01
PLXNA2	GDR	2.63	4.77E-02	1856	0.21	3.80E-01
AFTPH	GDR	2.62	4.83E-02	1867	0.24	2.79E-01
AIP	GDR	2.61	4.94E-02	1889	0.23	3.20E-01

Table S5. List of Gandal Down-Regulated (GDR) genes that were significantly weighted on top PLS1 component. For each gene PLS1, *z*-score represents the bootstrapped *Z*-score of the PLS weight. PLS1 p-value shows the significant level of the PLS1 *z*-score (*z*-test) after FDR-correction. PLS1 ranking shows the position of a gene in the PLS1 *z*-score list. Regional correlation and regional p-value illustrate the pairwise Pearson correlation between gene expression and regional values of schizotypy-related myelination (FDR-corrected).

Gene Name	Schizophrenia-related list	PLS1 Z-score	PLS1 Adjusted *P*-value	PLS1 Ranking out of 20,647	Inter-regional correlation	Inter-regional *P*-value
SLA	GUR	-6.11	1.16E-06	20639	-0.45	1.68E-02
YPEL1	GUR	-5.71	4.12E-06	20622	-0.41	3.41E-02
EPHX1	GUR	-5.70	4.12E-06	20619	-0.38	4.80E-02
FCGBP	GUR	-5.57	6.90E-06	20612	-0.39	4.15E-02
DPYSL3	GUR	-5.56	6.90E-06	20609	-0.35	7.47E-02
TMEM176B	GUR	-5.33	1.48E-05	20581	-0.37	5.68E-02
TMEM176A	GUR	-5.30	1.61E-05	20574	-0.43	2.62E-02
TLR4	GUR	-5.22	2.17E-05	20563	-0.44	2.19E-02
PEA15	GUR	-5.19	2.40E-05	20558	-0.36	7.04E-02
TTPA	GUR	-5.15	2.54E-05	20544	-0.35	7.52E-02
EDNRB	GUR	-4.98	4.29E-05	20497	-0.32	1.08E-01
PKIA	GUR	-4.97	4.45E-05	20495	-0.38	5.08E-02
ATP2B4	GUR	-4.97	4.57E-05	20494	-0.30	1.54E-01
TRIM24	GUR	-4.94	5.09E-05	20490	-0.48	9.96E-03
POLR2G	GUR	-4.93	5.31E-05	20487	-0.40	4.04E-02
PDLIM5	GUR	-4.90	5.83E-05	20480	-0.35	8.35E-02
PLXDC2	GUR	-4.89	6.15E-05	20478	-0.45	1.64E-02
PEG10	GUR	-4.83	7.53E-05	20460	-0.35	7.59E-02
SFT2D2	GUR	-4.81	8.19E-05	20453	-0.42	3.08E-02
UMPS	GUR	-4.77	9.05E-05	20437	-0.35	8.23E-02
C14orf132	GUR	-4.76	9.18E-05	20432	-0.40	3.84E-02
IL33	GUR	-4.71	1.11E-04	20415	-0.30	1.45E-01
MR1	GUR	-4.71	1.11E-04	20414	-0.38	4.80E-02
DPYD	GUR	-4.70	1.13E-04	20411	-0.36	7.33E-02
SLC1A4	GUR	-4.64	1.44E-04	20394	-0.33	1.05E-01
GPR6	GUR	-4.62	1.53E-04	20391	-0.41	3.18E-02
FCGR1A	GUR	-4.62	1.54E-04	20388	-0.41	3.17E-02
BNIP2	GUR	-4.57	1.87E-04	20381	-0.32	1.10E-01
KCNN3	GUR	-4.57	1.88E-04	20377	-0.29	1.73E-01
FABP7	GUR	-4.53	2.21E-04	20367	-0.34	8.75E-02
GPC4	GUR	-4.51	2.30E-04	20361	-0.34	8.87E-02
APOE	GUR	-4.49	2.46E-04	20355	-0.30	1.52E-01
CD99	GUR	-4.46	2.75E-04	20341	-0.34	8.92E-02
GPM6B	GUR	-4.42	3.18E-04	20325	-0.30	1.55E-01
TRAF3IP2	GUR	-4.36	3.83E-04	20302	-0.29	1.69E-01
TRHR	GUR	-4.34	4.17E-04	20292	-0.31	1.27E-01
CASP6	GUR	-4.34	4.19E-04	20291	-0.35	7.84E-02
ABCA1	GUR	-4.31	4.60E-04	20283	-0.35	7.50E-02
CARHSP1	GUR	-4.28	5.09E-04	20273	-0.31	1.38E-01
CPNE3	GUR	-4.22	6.31E-04	20244	-0.33	1.03E-01
FKBP14	GUR	-4.20	6.67E-04	20234	-0.24	2.74E-01
TAF7L	GUR	-4.17	7.35E-04	20217	-0.45	1.85E-02
FAM65B	GUR	-4.14	8.09E-04	20199	-0.25	2.61E-01
TMEM47	GUR	-4.13	8.15E-04	20197	-0.35	8.35E-02

FAM117A	GUR	-4.10	9.14E-04	20182	-0.28	1.82E-01
DNAJC3	GUR	-4.10	9.14E-04	20179	-0.32	1.16E-01
PLEKHF2	GUR	-4.07	9.85E-04	20156	-0.31	1.27E-01
TRIM27	GUR	-4.07	9.85E-04	20155	-0.29	1.60E-01
HIST2H2BE	GUR	-4.05	1.07E-03	20143	-0.35	8.37E-02
GNG12	GUR	-4.02	1.16E-03	20123	-0.31	1.26E-01
NQO1	GUR	-3.97	1.32E-03	20098	-0.33	9.94E-02
SCN9A	GUR	-3.97	1.32E-03	20097	-0.35	8.40E-02
SSPN	GUR	-3.93	1.53E-03	20074	-0.32	1.11E-01
SLC4A4	GUR	-3.91	1.64E-03	20061	-0.20	4.12E-01
SIDT2	GUR	-3.90	1.65E-03	20057	-0.31	1.31E-01
KAL1	GUR	-3.90	1.65E-03	20056	-0.31	1.31E-01
ERBB2	GUR	-3.90	1.66E-03	20054	-0.32	1.17E-01
GPC5	GUR	-3.85	1.92E-03	20013	-0.28	1.91E-01
SLC7A11	GUR	-3.84	1.97E-03	20006	-0.25	2.60E-01
TMED5	GUR	-3.80	2.21E-03	19980	-0.46	1.47E-02
HSDL2	GUR	-3.80	2.25E-03	19976	-0.29	1.72E-01
CYBRD1	GUR	-3.77	2.44E-03	19962	-0.27	2.18E-01
CETN3	GUR	-3.76	2.49E-03	19956	-0.34	8.43E-02
NPC2	GUR	-3.75	2.59E-03	19930	-0.29	1.62E-01
TTYH1	GUR	-3.74	2.63E-03	19927	-0.28	2.00E-01
AASS	GUR	-3.73	2.69E-03	19923	-0.27	2.15E-01
IFITM2	GUR	-3.68	3.15E-03	19869	-0.38	4.80E-02
TSPAN6	GUR	-3.66	3.28E-03	19857	-0.27	2.05E-01
SMPD2	GUR	-3.64	3.51E-03	19835	-0.24	2.94E-01
ROBO1	GUR	-3.59	4.02E-03	19811	-0.27	2.25E-01
CYP2J2	GUR	-3.56	4.46E-03	19791	-0.22	3.37E-01
ANGEL2	GUR	-3.53	4.91E-03	19768	-0.39	4.26E-02
PPAP2B	GUR	-3.53	4.94E-03	19766	-0.22	3.44E-01
ZZZ3	GUR	-3.50	5.24E-03	19746	-0.33	9.91E-02
ERLIN2	GUR	-3.47	5.70E-03	19709	-0.23	3.00E-01
NECAP2	GUR	-3.45	6.02E-03	19675	-0.23	3.00E-01
MYO10	GUR	-3.42	6.59E-03	19651	-0.20	4.04E-01
HK2	GUR	-3.41	6.75E-03	19644	-0.31	1.32E-01
MPP3	GUR	-3.40	6.79E-03	19640	-0.33	9.92E-02
MCM6	GUR	-3.40	6.84E-03	19636	-0.24	2.77E-01
GSTM2	GUR	-3.38	7.16E-03	19618	-0.21	3.72E-01
ACSS3	GUR	-3.36	7.67E-03	19602	-0.24	2.87E-01
CSTB	GUR	-3.34	8.25E-03	19581	-0.25	2.72E-01
NUPR1	GUR	-3.33	8.44E-03	19574	-0.27	2.18E-01
CFLAR	GUR	-3.29	9.28E-03	19541	-0.30	1.58E-01
GABRB1	GUR	-3.27	9.95E-03	19514	-0.24	2.79E-01
SH3GLB1	GUR	-3.25	1.02E-02	19498	-0.25	2.68E-01
PTPN9	GUR	-3.25	1.03E-02	19496	-0.26	2.34E-01
SELENBP1	GUR	-3.23	1.09E-02	19466	-0.27	2.16E-01
SERP1	GUR	-3.22	1.12E-02	19456	-0.28	1.79E-01

LYN	GUR	-3.20	1.16E-02	19438	-0.27	2.09E-01
HEPH	GUR	-3.20	1.16E-02	19436	-0.17	5.18E-01
TMEM43	GUR	-3.18	1.24E-02	19416	-0.28	1.93E-01
OTUD4	GUR	-3.18	1.24E-02	19414	-0.24	2.77E-01
PTPN13	GUR	-3.17	1.26E-02	19405	-0.25	2.61E-01
HMGA2	GUR	-3.16	1.30E-02	19385	-0.29	1.76E-01
STK17B	GUR	-3.14	1.36E-02	19359	-0.21	3.86E-01
KAT2B	GUR	-3.13	1.39E-02	19348	-0.23	3.14E-01
MT1H	GUR	-3.13	1.40E-02	19344	-0.24	2.89E-01
ZC3HAV1	GUR	-3.12	1.42E-02	19335	-0.26	2.47E-01
PRKG1	GUR	-3.11	1.46E-02	19320	-0.20	3.93E-01
ANGPTL4	GUR	-3.10	1.48E-02	19310	-0.20	3.98E-01
WFS1	GUR	-3.10	1.49E-02	19308	-0.21	3.73E-01
ZNF253	GUR	-3.10	1.49E-02	19304	-0.29	1.71E-01
PLEKHO2	GUR	-3.07	1.59E-02	19272	-0.20	4.09E-01
PDGFRL	GUR	-3.07	1.61E-02	19267	-0.33	1.01E-01
TAF10	GUR	-3.05	1.68E-02	19239	-0.24	2.99E-01
IKZF5	GUR	-3.05	1.69E-02	19237	-0.25	2.60E-01
RARRES3	GUR	-3.04	1.70E-02	19232	-0.23	3.08E-01
BIRC3	GUR	-3.04	1.73E-02	19221	-0.24	2.85E-01
GDPD2	GUR	-3.03	1.74E-02	19218	-0.25	2.72E-01
TBL1X	GUR	-3.03	1.76E-02	19214	-0.26	2.45E-01
ADCYAP1R1	GUR	-3.03	1.77E-02	19209	-0.23	3.07E-01
PAK2	GUR	-3.01	1.83E-02	19194	-0.27	2.13E-01
TIMP1	GUR	-3.00	1.87E-02	19180	-0.23	3.00E-01
CADM1	GUR	-3.00	1.89E-02	19176	-0.22	3.55E-01
ZNF471	GUR	-2.99	1.94E-02	19166	-0.24	2.89E-01
AQP4	GUR	-2.99	1.96E-02	19163	-0.24	2.78E-01
TPP1	GUR	-2.98	2.00E-02	19154	-0.27	2.16E-01
PAICS	GUR	-2.98	2.00E-02	19152	-0.27	2.09E-01
PRSS23	GUR	-2.98	2.01E-02	19150	-0.19	4.52E-01
TMED10	GUR	-2.98	2.01E-02	19147	-0.22	3.28E-01
ZNF350	GUR	-2.97	2.05E-02	19128	-0.20	3.96E-01
SCGB1D2	GUR	-2.97	2.05E-02	19127	-0.22	3.45E-01
CP	GUR	-2.95	2.11E-02	19115	-0.27	2.13E-01
MT2A	GUR	-2.95	2.14E-02	19106	-0.21	3.58E-01
NFE2	GUR	-2.93	2.21E-02	19083	-0.21	3.63E-01
SIRT1	GUR	-2.93	2.23E-02	19078	-0.22	3.38E-01
FAM107A	GUR	-2.92	2.30E-02	19060	-0.19	4.22E-01
SLC1A2	GUR	-2.91	2.31E-02	19053	-0.16	5.45E-01
IGF2BP2	GUR	-2.91	2.34E-02	19049	-0.23	3.04E-01
REST	GUR	-2.90	2.40E-02	19042	-0.23	3.23E-01
CDH20	GUR	-2.90	2.42E-02	19038	-0.25	2.60E-01
STOM	GUR	-2.89	2.43E-02	19030	-0.22	3.29E-01
ITGB8	GUR	-2.89	2.44E-02	19029	-0.16	5.56E-01
C1R	GUR	-2.88	2.54E-02	19019	-0.22	3.27E-01

FOXO1	GUR	-2.87	2.57E-02	19013	-0.27	2.06E-01
YAP1	GUR	-2.85	2.73E-02	18987	-0.20	4.05E-01
ADD3	GUR	-2.84	2.82E-02	18973	-0.27	2.22E-01
GPR56	GUR	-2.83	2.83E-02	18968	-0.19	4.43E-01
DUS4L	GUR	-2.83	2.84E-02	18956	-0.31	1.38E-01
MTHFD2L	GUR	-2.83	2.84E-02	18957	-0.26	2.35E-01
CSGALNACT1	GUR	-2.82	2.89E-02	18952	-0.19	4.52E-01
MLC1	GUR	-2.82	2.89E-02	18950	-0.20	4.19E-01
CTBP2	GUR	-2.82	2.91E-02	18947	-0.17	4.92E-01
ABI1	GUR	-2.82	2.94E-02	18939	-0.20	4.00E-01
CYP7B1	GUR	-2.81	2.95E-02	18937	-0.22	3.44E-01
SERPING1	GUR	-2.81	2.98E-02	18935	-0.31	1.39E-01
ARID1A	GUR	-2.80	3.03E-02	18926	-0.23	3.20E-01
AGT	GUR	-2.78	3.15E-02	18895	-0.28	2.00E-01
METTL7A	GUR	-2.78	3.15E-02	18893	-0.16	5.37E-01
TP53BP2	GUR	-2.78	3.18E-02	18887	-0.19	4.38E-01
FKBP10	GUR	-2.75	3.40E-02	18845	-0.25	2.57E-01
KLF7	GUR	-2.75	3.42E-02	18837	-0.21	3.72E-01
TIMP3	GUR	-2.75	3.43E-02	18831	-0.21	3.82E-01
MT1G	GUR	-2.74	3.48E-02	18820	-0.20	4.06E-01
GSTM4	GUR	-2.74	3.50E-02	18817	-0.23	3.14E-01
NAGA	GUR	-2.72	3.63E-02	18786	-0.23	3.12E-01
NTRK2	GUR	-2.71	3.69E-02	18769	-0.21	3.86E-01
TLR2	GUR	-2.70	3.80E-02	18752	-0.22	3.43E-01
SLC2A5	GUR	-2.69	3.84E-02	18737	-0.23	3.02E-01
PREX2	GUR	-2.68	3.91E-02	18720	-0.24	2.91E-01
RFX3	GUR	-2.68	3.91E-02	18714	-0.21	3.56E-01
SMAD1	GUR	-2.66	4.08E-02	18684	-0.20	3.94E-01
PIR	GUR	-2.66	4.09E-02	18681	-0.20	4.06E-01
TMPRSS5	GUR	-2.65	4.22E-02	18665	-0.19	4.23E-01
ZNF480	GUR	-2.64	4.27E-02	18659	-0.23	3.22E-01
GJA1	GUR	-2.63	4.44E-02	18641	-0.18	4.76E-01
ALDH1L1	GUR	-2.61	4.57E-02	18627	-0.18	4.77E-01
KIAA1598	GUR	-2.60	4.67E-02	18613	-0.22	3.37E-01
BTG1	GUR	-2.60	4.69E-02	18611	-0.28	1.87E-01
CBFB	GUR	-2.60	4.69E-02	18608	-0.21	3.63E-01
ALDH6A1	GUR	-2.60	4.73E-02	18603	-0.20	4.00E-01
H2AFJ	GUR	-2.59	4.77E-02	18596	-0.23	3.25E-01
ROM1	GUR	-2.59	4.77E-02	18595	-0.26	2.44E-01

Table S6. List of Gandal Up-Regulated (GUR) genes that were significantly weighted on bottom PLS1 component. For each gene in PLS1, *z*-score represents the bootstrapped *Z*-score of the PLS weight. PLS1 p-value shows the significance level of the PLS1 *z*-score (*z*-test) after FDR-correction. PLS1 ranking shows the position of the gene in the PLS1 *z*-score list. Regional correlation and regional p-value illustrate the pairwise Pearson correlation between gene expression and regional values of schizotypy-related myelination (FDR-corrected).

Supplementary References

1. Ziegler G, Moutoussis M, Hauser TU, Fearon P, Bullmore ET, Goodyer IM, *et al.* (2019): Childhood socio-economic disadvantage predicts reduced myelin growth across adolescence and young adulthood. *bioRxiv*. 589713.

2. Dale AM, Fischl B, Sereno MI (1999): Cortical surface-based analysis. I. Segmentation and surface reconstruction. *Neuroimage*. 9: 179–194.

3. Dale AM, Sereno MI (1993): Improved Localizadon of Cortical Activity by Combining EEG and MEG with MRI Cortical Surface Reconstruction: A Linear Approach. *J Cogn Neurosci*. 5.

4. Desikan RS, Ségonne F, Fischl B, Quinn BT, Dickerson BC, Blacker D, *et al.* (2006): An automated labeling system for subdividing the human cerebral cortex on MRI scans into gyral based regions of interest. *Neuroimage*. 31: 968–980.

5. Yarkoni T, Poldrack R, Nichols T, Van Essen D, Wager T (2011): NeuroSynth: a new platform for large-scale automated synthesis of human functional neuroimaging data. *Front Neuroinformatics Conf Abstr 4th INCF Congr Neuroinformatics*. p doi: 10.3389/conf.fninf.2011.08.00058.

6. Hawrylycz MJ, Lein ES, Guillozet-Bongaarts AL, Shen EH, Ng L, Miller JA, *et al.* (2012): An anatomically comprehensive atlas of the adult human brain transcriptome. *Nature*. 489:391-39.

7. Arloth J, Bader DM, Röh S, Altmann A (2015): Re-Annotator: Annotation pipeline for microarray probe sequences. *PLoS One*. 10. doi: 10.1371/journal.pone.0139516.

8. Miller JA, Cai C, Langfelder P, Geschwind DH, Kurian SM, Salomon DR, Horvath S (2011): Strategies for aggregating gene expression data: The collapseRows R function. *BMC Bioinformatics*. 12: 322.

9. Arnatkevičiūtė A, Fulcher BD, Fornito A (2019): A practical guide to linking brain-wide gene expression and neuroimaging data. *Neuroimage*. 189: 353–367.

10. Toker L, Mancarci BO, Tripathy S, Pavlidis P (2018): Transcriptomic Evidence for Alterations in Astrocytes and Parvalbumin Interneurons in Subjects With Bipolar Disorder and Schizophrenia. *Biol Psychiatry*. 84: 787–796.

11. Romme IAC, de Reus MA, Ophoff RA, Kahn RS, van den Heuvel MP (2016): Connectome Disconnectivity and Cortical Gene Expression in Patients With Schizophrenia. *Biol Psychiatry*. 1–8.

12. Gandal MJ, Haney JR, Parikshak NN, Leppa V, Ramaswami G, Hartl C, *et al.* (2018): Shared molecular neuropathology across major psychiatric disorders parallels polygenic overlap. *Science (80-)*. 359: 693–697.

13. Szklarczyk D, Franceschini A, Wyder S, Forslund K, Heller D, Huerta-Cepas J, *et al.* (2015): STRING v10: Protein-protein interaction networks, integrated over the tree of life. *Nucleic Acids Res*. 43: D447–D452.

14. Fromer M, Roussos P, Sieberts SK, Johnson JS, Kavanagh DH, Perumal TM, *et al.* (2016): Gene expression elucidates functional impact of polygenic risk for schizophrenia. *Nat Neurosci*. 19: 1442-1.
